# Ammonium metavanadate (NH_4_VO_3_): a highly efficient and eco-friendly catalyst for one-pot synthesis of pyridines and 1,4-dihydropyridines

**DOI:** 10.1038/s41598-022-17378-7

**Published:** 2022-08-11

**Authors:** Jamal Rahimi, Maryam Niksefat, Marzieh Heidari, Mehdi Naderi, Hadis Abbasi, Mohammad Tajik Ijdani, Ali Maleki

**Affiliations:** 1 Catalysts and Organic Synthesis Research Laboratory, Department of Chemistry, Iran University of Science and Technology, Tehran, 16846-13114 Iran; 2 Department of Chemistry, Rutgers University, 73 Warren Street, Newark, NJ 07102 USA

**Keywords:** Chemistry, Catalysis, Green chemistry, Organic chemistry

## Abstract

In this study, we reported the ammonium metavanadate (NH_4_VO_3_) as an efficient, cost-effective, and mild catalyst for the synthesis of substituted pyridines via a one-pot pseudo four-component reaction. Furthermore, we investigated Hantzsch 1,4-dihydropyridines (1,4-DHPs) synthesis and oxidation of 1,4-DHPs to their corresponding pyridines. The present approach offers a rapid methodology for accessing various pyridines with broad functional group tolerance and good yields using NH_4_VO_3_ catalyst as a green catalyst.

## Introduction

**Figure 1:**
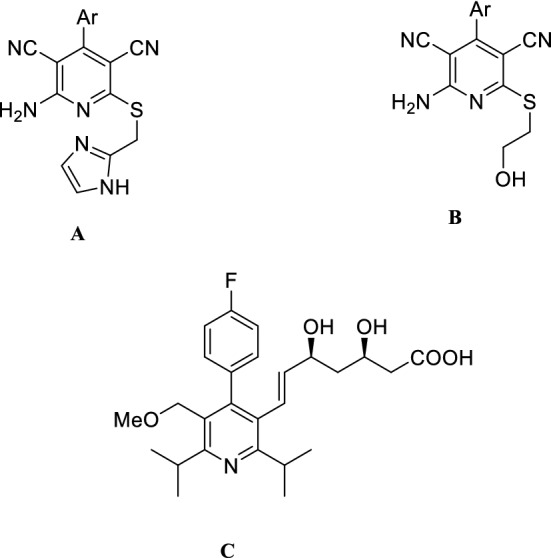
Substituted pyridines as privileged structures.

For several decades nitrogen-containing six-membered heterocyclic compounds have attracted the interest of synthetic organic chemists due to their pharmaceutical and biological properties. Among the nitrogen heterocycles, pyridine derivatives are well known as calcium channel blockers and exhibit therapeutic effects, such as vasodilator, bronchodilator, geroprotective, hepatoprotective, neuroprotective, and anti-tumor activity^1–4^. For example, there are many pharmaceutical pyridine compounds (Fig. 1) such as (**A)** and (**B)**, as selective modulators of human adenosine receptors implicated in asthma, Parkinson’s disease, epilepsy, kidney disease, and cancer, as well as cerivastatin (**C)** for the treatment of atherosclerosis and other coronary diseases^5–8^. Pyridine derivatives are not only privileged scaffolds for drug discovery but also used as building blocks reagents in organic synthesis and ligands in coordination chemistry^9^. Due to their importance, the development of novel synthetic methods for the preparation of pyridine derivatives is of interest^10,11^.

The traditional so-called Hantzsch synthesis of 1,4-DHPs includes one-pot cyclocondensation of a *β*-ketoester with an aldehyde and a nitrogen source, which occurs either in acetic acid at room temperature or by refluxing in alcohols; this protocol has some drawbacks such as prolonged reaction times and low yields^12^. Therefore, numerous modifications have been made to the original Hantzsch reaction, such as using microwave radiation^13,14^, ionic liquid^15^, SiO_2_/NaHSO_4_^16^, metal triflates^17^, I_2_^18^, ceric ammonium nitrate (CAN)^19^ and ZnO^20^.

Recently, the oxidation of 1,4-DHPs was successfully carried out by using various oxidants, such as peroxydisulfate-Co(II)^21^, silica-modified sulfuric acid/NaNO_2_^22^, Co-naphthenate^23^, KBrO_3_/SnCl_4_.5H_2_O^24^, MnO_2_^25^, silica chromate^26^, urea- hydrogen peroxide catalyzed by molecular iodine^27^, b-cyclodextrin^28^, silica-sulfuric acid and Al(NO_3_)_3_·9H_2_O or Fe(NO_3_)_3_·9H_2_O^29^.

In recent years, the application of the bifunctional solid acid/ noble metal Pd/C/K-10 catalyst was reported for the one-pot synthesis of pyridine derivatives^30,31^. In addition, Khaskel and Barman reported the one-pot synthesis of pyridines in ethanol by benzyltrimethylammoniumfluoride hydrate (BTMAFH) and K_2_S_2_O_8_^32^. Ghosh et al. reported the direct synthesis of pyridine derivatives using visible light in aqueous media catalyzed by non-ionic surfactant Triton-X-100^33^. Although, many of the reported methods for synthesis of pyridine derivatives offer distinct benefits, some of them still have some drawbacks, such as long reaction times, expensive reagents, harsh conditions, low product yields, tedious work-up, and by-products formation.

**Figure 2:**
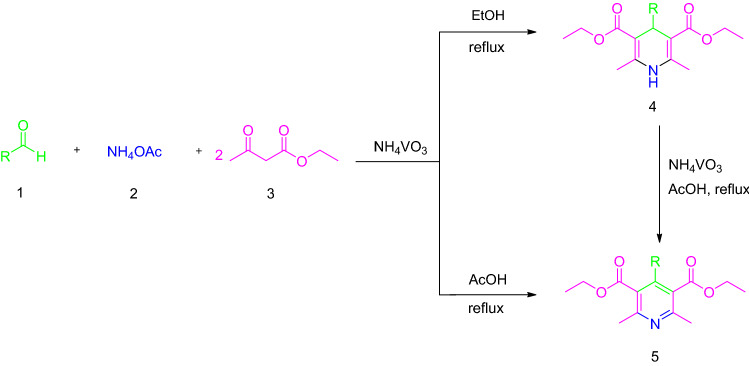
One-pot synthesis of pyridines, 1,4-DHPs, and the oxidation aromatization of 1,4-DHPs to the corresponding pyridines.

Hence, the development of a new procedure for the one-pot synthesis of pyridine derivatives would be highly desirable. Recently, NH_4_VO_3_ has been utilized as an inorganic acid and economical catalyst in organic synthesis^34–36^. Furthermore, to the best of our knowledge the use of NH_4_VO_3_ in the synthesis of pyridine derivatives has been never reported before. In continuation of our previous works on the introduction of new catalysts in organic synthesis^37–43^, herein, we report the use of NH_4_VO_3_ without any post-modification as an efficient, inexpensive, and eco-friendly catalyst for the synthesis of substituted pyridines via one-pot pseudo four-component reaction, including a combination of the Hantzsch synthesis and the subsequent oxidation step for the first time (Fig. 2).

## Experimental

### General

All solvents, chemicals, and reagents were purchased from Merck, Fluka, and Sigma-Aldrich chemical companies. Melting points were measured with an Electrothermal 9100 apparatus and are uncorrected. FT-IR spectra were obtained over 400–4000 cm^−1^ with a Shimadzu IR-470 spectrometer using KBr pellets. ^1^H-NMR and ^13^C-NMR spectra were recorded by a Bruker Avance DRX500 spectrometer. All the synthesized products were known, and the structure of the isolated products was confirmed by previously reported data.

### General procedure for one-pot synthesis of pyridines

A mixture of an aldehyde **1** (1.0 mmol), ammonium acetate **2** (2.0 mmol), ethyl acetoacetate **3** (2.0 mmol), and ammonium metavanadate (NH_4_VO_3_) (117.0 mg) in 3.0 mL acetic acid was stirred under reflux condition for the appropriate time (Table 7). After completion of the reaction, as indicated by thin-layer chromatography (TLC), the catalyst (NH_4_VO_3_) was separated by filtration. Then, products afforded by evaporation of the solvent, and recrystallized from diethyl ether to give the pure desired pyridines (**5**).

### General procedure for preparation of 1,4-DHPs

A mixture of an aldehyde **1** (1.0 mmol), ammonium acetate **2** (2.0 mmol), ethyl acetoacetate **3** (2.0 mmol), and ammonium metavanadate (NH_4_VO_3_) (15.0 mg) in 3.0 mL ethanol was stirred under reflux condition for the appropriate time (Table 2). After completion of the reaction, as indicated by thin-layer chromatography (TLC), the catalyst (NH_4_VO_3_) was separated by filtration, washed with ethanol, and reused five times in other fresh reactions without a considerable loss of activity. Then, products (**4**) are afforded by evaporation of the solvent, followed by recrystallization from ethanol.

### General procedure for oxidative aromatization of 1,4-DHPs

To a solution of 1,4-DHPs **4** (1.0 mmol) in 3.0 mL of acetic acid, ammonium metavanadate (NH_4_VO_3_) (117.0 mg) was added. The resulting mixture was refluxed for an appropriate time (Table 5). After completion of the reaction (monitored by TLC), the mixture was cooled to room temperature and the catalyst was filtered off. Then the filtrate was evaporated and recrystallized from diethyl ether to give the pure desired pyridines (**5**).

### Spectral data

*Diethyl 4-(4-methoxyphenyl)-2,6-dimethyl-3,5-pyridinedicarboxylate* **(5d)**: FT-IR (KBr: υ/cm^−1^): 2985, 2929, 1724, 1558, 1510, 1488, 1294, 1232, 1107, 1045, 860, 792; ^1^H NMR (500 MHz, CDCl_3_): *δ*_H_ (ppm) = 1.08 (t, 6H, *J* = 7.1 Hz, CH_3_), 2.68 (s, 6H, CH_3_), 3.92(s, 3H, OCH_3_) 4.14 (q, 4H, *J* = 7.1 Hz, CH_2_), 6.99 (d, 2H, *J* = 8.7 Hz, H-Ar), 7.29 (d, 2H, *J* = 8.7 Hz, H-Ar).

*Diethyl 4-(4-bromophenyl)-2,6-dimethyl-3,5-pyridinedicarboxylate* **(5e)**: FT-IR (KBr: υ/cm^−1^): 2981, 2931, 1726, 1556, 1488, 1446, 1292, 1232, 1211, 1103, 1043, 860, 829 ; ^1^H NMR (500 MHz, CDCl_3_): *δ*_H_ (ppm) = 0.97 (t, 6H, *J* = 7.1 Hz, CH_3_), 2.58 (s, 6H, CH_3_), 4.03 (q, 4H, *J* = 7.1 Hz, CH_2_), 7.12 (d, 2H, *J* = 8.4 Hz, H-Ar), 7.50 (d, 2H, *J* = 8. 4 Hz, H-Ar).

*Diethyl 4-(4-chlorophenyl)-2,6-dimethyl-3,5-pyridinedicarboxylate* **(5f.)**: FT-IR (KBr: υ/cm^−1^): 2983, 1724, 1554, 1292, 1232, 1097, 1043, 860, 665; ^1^H NMR (500 MHz, DMSO): *δ*_H_ (ppm) = 0.97 (t, 6H, *J* = 7.1 Hz, CH_3_), 2.59 (s, 6H, CH_3_), 4.10 (q, 4H, *J* = 7.1 Hz, CH_2_), 7.27 (d, 2H, *J* = 8. 4 Hz, H-Ar), 7.61 (d, 2H, *J* = 8. 7 Hz, H-Ar).

*Diethyl 2,6-dimethyl-4-(thiophen-2-yl)pyridine-3,5-dicarboxylate* **(5 m)**: FT-IR (KBr: υ/cm^−1^): 2981, 2933, 1728, 1558, 1444, 1288, 1234, 1099, 1041, 860, 705; ^1^H NMR (500 MHz, CDCl_3_): *δ*_H_ (ppm) = 1.17 (t, 6H, *J* = 7.1 Hz, CH_3_), 2.68 (s, 6H, CH_3_), 4.23 (q, 4H, *J* = 7.1 Hz, CH_2_), 7.15 (bs, 2H, H-Ar), 7.50 (bs, 1H, H-Ar).

*4-(4-methoxy-phenyl)-2,6-dimethyl-1,4-dihydro-pyridine-3,5-dicarboxylic acid diethyl ester* **(4d)**: FT-IR (KBr: υ/cm^−1^): 682, 838, 1026, 1209, 1496, 1650, 1689, 2974, 3340; ^1^H NMR (500 MHz, CDCl_3_): *δ*_H_ (ppm) = 7.31 (d, 2H, *J* = 8.5 Hz, H-Ar), 1.33 (t, 6H, *J* = 7.1 Hz, CH_3_), 6.86 (d, 2H, *J* = 8.5 Hz, H-Ar), 6.01 (s, 1H, NH), 5.04 (s, 1H, CH), 4.20 (m, 4H, CH_2_), 3.86 (s, 3H, OCH_3_), 2.41 (s, 6H, CH_3_).

*4-(4-bromo-phenyl)-2,6-dimethyl-1,4-dihydro-pyridine-3,5-dicarboxylic acid diethyl ester* **(4e):** FT-IR (KBr: υ/cm^−1^): 780, 1012, 1217, 1377, 1488, 1652, 1693, 2989, 3357; ^1^H NMR (500 MHz, DMSO): *δ*_H_ (ppm) = 8.92 (s, 1H, NH), 7.22–7.32 (m, 4H, H-Ar), 4.90 (s, 1H, CH), 4.90 (s, 1H, CH), 4.16–4.24 (m, 4H, CH_2_, broad), 2.32 (m, 6H, CH_3,_ broad), 1.18 (s, 6H, CH_3_); ^13^C NMR (125 MHz, DMSO): *δ*_C_ (ppm) = 166.8, 147.1, 145.6, 130.4, 129.2, 127.8, 101.5, 59.0, 38,5, 18.2, 14.1.

*4-(4-chloro-phenyl)-2,6-dimethyl-1,4-dihydro-pyridine-3,5-dicarboxylic acid diethyl ester* **(4f.):** FT-IR (KBr: υ/cm^−1^): 1213, 1371, 1487, 1652, 1695, 3357; ^1^H NMR (500 MHz, DMSO): *δ*_H_ (ppm) = 8.92 (s, 1H, NH), 7.22–7.32 (m, 4H, H-Ar), 4.90 (s, 1H, CH), 4.04 (m, 4H, CH_2_, broad), 2.32 (s, 6H, CH_3_), 1.18 (s, 6H, CH_3_); ^13^C NMR (125 MHz, DMSO): *δ*_C_ (ppm) = 166.8, 14.1, 147.1, 145.6, 130.4, 129.2, 127.8, 101.5, 59.0, 38,5, 18.2.

*2,6-dimethyl-4-(3-nitro-phenyl)-1,4-dihydro-pyridine-3,5-dicarboxylic acid diethyl ester* **(4j)**: FT-IR (KBr: υ/cm^−1^): 1118, 1213, 1348, 1487, 1647, 1704, 2987, 3346; ^1^H NMR (500 MHz, DMSO): *δ*_H_ (ppm) = 8.94 (s, 1H, NH), 7.47 (d, 2H, *J* = 8. 4 Hz, H-Ar), 7.17 (d, 2H, *J* = 8. 4 Hz, H-Ar), 4.90 (s, 1H, CH), 4.01–4.11 (m, 4H, CH_2_), 2.33 (s, 6H, CH_3_), 1.20 (t, 6H, *J* = 7.1 Hz, CH_3_).

*2,6-dimethyl-4-(thiophen-2-yl)-1,4-dihydro-pyridine-3,5-dicarboxylic acid diethyl ester* **(4 m)**: FT-IR (KBr: υ/cm^−1^): 719, 1124, 1209, 1299, 1371, 1487, 1652, 1695, 2985, 3344; ^1^H NMR (500 MHz, CDCl_3_): *δ*_H_ (ppm) = 7.47 (dd, 1H, *J* = 1.2 Hz, *J* = 3.9 Hz, H-Ar), 6.90–6.97 (m, 2H), 5.97 (s, 1H, NH), 5.46 (s, 1H, CH), 4.25–4.32 (m, 4H, CH_2_), 2.45 (s, 6H, CH_3_), 1.38 (t, 6H, *J* = 7.1 Hz, CH_3_).

## Results and discussion

**Figure 3:**
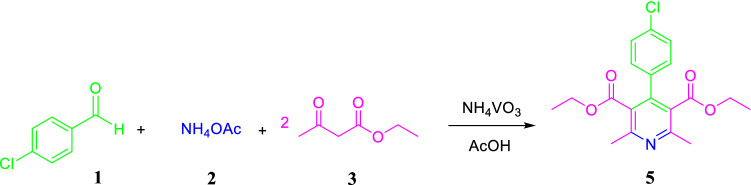
One-pot synthesis of pyridine derivatives.

Regarding the fact that the one-pot approach to the synthesis of substituted pyridines through Hantzsch synthesis is hardly carried out and there are only a few literatures reported in this field. Hence, the efficiency of ammonium metavanadate (NH_4_VO_3_) was investigated in the one-pot synthesis of pyridine derivatives. In an initial attempt, the condensation of 4-chlorobenaldehyde (1.0 mmol) with ethyl acetoacetate (2.0 mmol) and ammonium acetate (2.0 mmol) as a model reaction (Fig. 3) was examined in the presence of different catalytic amounts of NH_4_VO_3_ in acetic acid for the one-pot synthesis of pyridine derivatives. Surprisingly, when NH_4_VO_3_ was used as the catalyst in acetic acid under reflux conditions, the reaction went to completion in 10 min and 96% of the pyridine (product **8f.**) was isolated as the desired product.

To optimize the amount of catalyst and reaction conditions for the one-pot synthesis of pyridines, the model reaction was examined in acetic acid (Table 1). As shown in Table 1, the best results were achieved when the reaction was carried out in the presence of 117.0 mg of NH_4_VO_3_ as the catalyst in acetic acid under reflux conditions (entry 1, Table 1). Increasing the amount of catalyst (117.0–120.0 mg) did not improve the yield of the desired product (entries 1–5, Table 1). In the absence of NH_4_VO_3_ catalyst, the reaction was not successful (entry 11, Table 1).

**Table 1:** Screening of the amount of catalyst and reaction conditions for the one-pot synthesis of pyridines.

Entry	Solvent	Time(min)	Amount of catalyst (mg)	Temperature (°C)	Yield^a^ (%)
1	Acetic acid	10	117	Reflux	96
2	Acetic acid	10	117	Reflux	96^b^
3	Acetic acid	10	117	Reflux	96^c^
4	Acetic acid	10	120	Reflux	96
5	Acetic acid	10	180	Reflux	96
6	Acetic acid	60	29	Reflux	67
7	Acetic acid	60	58	Reflux	73
8	Acetic acid	60	88	Reflux	85
9	Acetic acid	60	116	Reflux	90
10	Acetic acid	60	117	r.t	65
11	Acetic acid	60	–	Reflux	0

Reaction conditions: 4-chlorobenaldehyde (1.0 mmol), ethyl acetoacetate (2.0 mmol), ammonium acetate (2.0 mmol), AcOH (3.0 mL), under air condition. ^a^Isolated yields. ^b^Under N_2_ atmosphere. ^c^Under O_2_ atmosphere.

**Figure 4:**
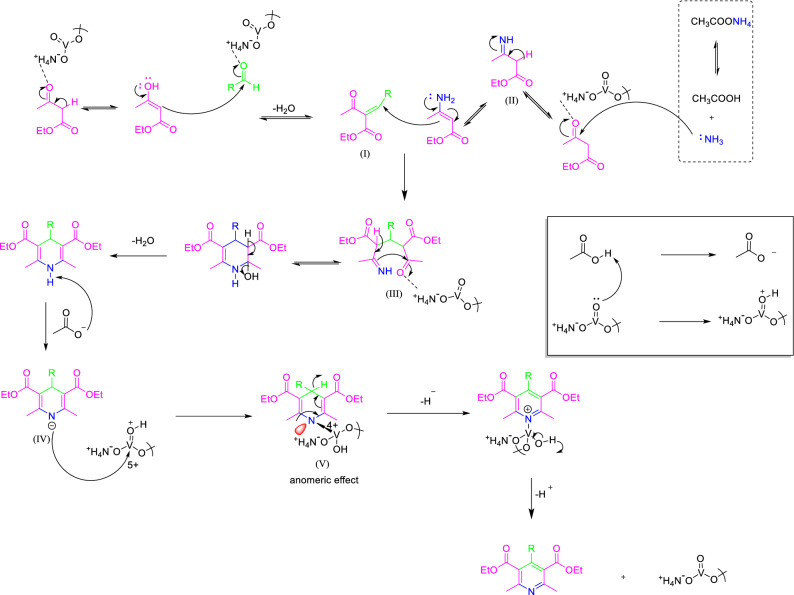
Proposed mechanism for the one-pot synthesis of pyridines by NH_4_VO_3_.

After optimizing the reaction conditions, to explore the scope of the reaction, a series of pyridine derivatives were synthesized by various aldehydes including both electron-donating and electron-withdrawing substituents (Table 7). All the aldehydes with both electron-withdrawing groups and electron-donating groups reacted very well, giving high yields of the desired products in short reaction times. Based on the results, we propose a plausible mechanism for the one-pot synthesis of pyridines (Fig. 4). This mechanistic pathway includes a combination of the Hantzsch synthesis and the subsequent oxidation step. First, the ammonium (NH_4_^+^) group in the structure of NH_4_VO_3_ activates the carbonyl functional groups of aldehyde and ethyl acetoacetate by hydrogen bonding. Therefore, it increases the carbonyl activity to Knoevenagel condensation with enol form of ethyl acetoacetate to give the corresponding Knoevenagel intermediate (**I**). In the next step, the reaction of the second molecule of ethyl acetoacetate with ammonium acetate gives the imine intermediate (**II**). The Michael addition of **I** with enamine form of **II** occurs to form intermediate **III**, which is activated through hydrogen bonding from NH_4_VO_3_ to facilitate cyclization and elimination of water, affording the desired 1,4-DHP derivatives. In continue, acetic acid using NH_4_VO_3_ as a catalyst is converted into acetate ion which is leading to an acid–base reaction with 1,4-DHPs. In the following, the negative charge of nitrogen of intermediate (**IV**) binds with the vacant “d” orbital of transition metal vanadium to achieve the stable oxidation state of vanadium. The last step might be progressed through unusual hydride transfer and H_2_ releasing from (**V**). For proving this opinion, the reaction was evaluated under a nitrogen atmosphere (entry 2, Table 1). The results show that the oxidation reaction progressed in an atmosphere of nitrogen similar to the air or oxygen atmosphere condition (entries 1–3, Table 1). Due to electron-donating from the nitrogen lone pairs into the anti-bonding orbital of C–H (s^*^_C–H_ orbital), the C–H bond is easily broken by reaction with a proton to afford molecular hydrogen. This phenomenon has been known as the anomeric effect.

Although there are a few literatures that reported on the direct approach for the one-pot synthesis of pyridines, this method is superior to the earlier methods in terms of yields, reaction time, and mild reaction conditions (Table 2).

**Table 2:** Comparison of different catalysts in the one-pot synthesis of pyridine derivatives.

Entry	Catalyst	Condition	Time (min)	Yield^a^ (%)	References
1	FeCl_3_ (1 mmol)	H_2_O/reflux	240	55	^44^
2	Triton-X-100 (10 mol%) + K_2_S_2_O_8_ (1 mmol)	H_2_O/RT	150	82	^33^
3	FeWO_4_ (20 mol%)	Acetic acid/80 (°C)	120	83	^32^
4	Catalyst-free	Solvent-free/20 (°C)	72 h	6.5	^45^
5	Pd/C/K-10 (200.0 mg Pd/C + 500.0 mg K-10)	MW/130 (°C)	90	75	^29^
6	NH_4_VO_3_ (117.0 mg)	Acetic acid/reflux	15	96	This work

Reaction conditions: 4-chlorobenzaldehyde (1.0 mmol), ethyl acetoacetate (2.0 mmol), ammonium acetate (2.0 mmol). ^a^Isolated yields.

**Figure 5:**
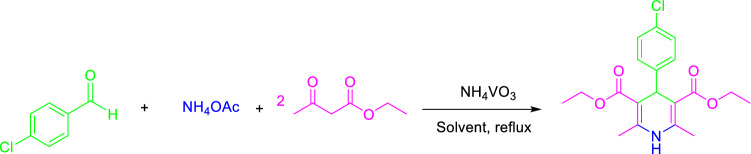
Hantzsch synthesis of 1,4-DHPs catalyzed by NH_4_VO_3_.

To further confirm the possible mechanism, we also examined the efficiency of NH_4_VO_3_ as a catalyst for the one-pot synthesis of 1,4-DHPs. To optimize the reaction conditions. The condensation of 4-chlorobenaldehyde (1.0 mmol) with ethyl acetoacetate (2.0 mmol) and ammonium acetate (2.0 mmol) as a model reaction (Fig. 5) was chosen and the effect of different catalytic amounts of NH_4_VO_3_ in a wide variety of solvents and under reflux condition were investigated (Table 3).

**Table 3:** Optimization of the NH_4_VO_3_ catalyzed model reaction for the synthesis of Hantzsch 1,4-DHPs.

Entry	Solvent	Time(min)	Amount of catalyst (mg)	Temperature (°C)	Yield^a^ (%)
1	Dimethyl sulfoxide	20	15	Reflux	75
2	Polyethylene glycol	45	15	Reflux	90
3	Dimethylformamide	20	15	Reflux	45
4	Tetrahydrofuran	45	15	Reflux	37
5	Acetonitrile	20	15	Reflux	85
6	Water	20	15	Reflux	55
7	Ethanol	20	15	Reflux	93
8	Ethanol	45	15	Reflux	93
9	Ethanol	45	15	r. t	65
10	Ethanol	20	18	Reflux	93
11	Ethanol	20	21	Reflux	93
12	Ethanol	20	23	Reflux	93
13	Ethanol	20	14	Reflux	85
14	Ethanol	45	13	Reflux	70
15	Ethanol	45	12	Reflux	58
16	Ethanol	45	–	Reflux	31

Reaction conditions: 4-chlorobenaldehyde (1.0 mmol), ethyl acetoacetate (2.0 mmol), ammonium acetate (2.0 mmol), solvent (3.0 mL). ^a^Isolated yields.

In the absence of NH_4_VO_3_ as the catalyst, the reaction proceeded slowly with a low yield (entry 16, Table 3). As seen in Table 3 (entries 7–12) using 15.0–23.0 mg of the catalyst (NH_4_VO_3_) showed higher activity for the synthesis of 1,4-DHPs. However, when the amount of catalyst increased to 18.0–23.0 mg (entries 10–12, Table 3) the yield of the desired product (93%) did not improve. Among the investigated solvents, ethanol is the best choice with its short reaction time, high yield, cheapness, and being environmentally friendly for this reaction. According to the results in Tables (1,3), it is obvious that in the absence of acetic acid and using other solvents the 1,4-DHPs form as the desired products. After optimizing the reaction conditions, the effect of substitution on the aldehydes has also been studied. As shown in Table 7 all the aromatic aldehydes with both electron-withdrawing groups and electron-donating groups reacted very well, giving high yields of the desired products. As expected substituted aldehydes with electron-withdrawing groups require a shorter reaction time in comparison to those with electron-donating groups.

Moreover, the catalytic activity of the NH_4_VO_3_ for the synthesis of 1,4-DHPs was compared to the other reported catalysts in Table 4.

**Table 4:** Comparison of the efficiency of NH_4_VO_3_ with other catalysts for synthesizing 1,4-DHP (1f.).

Entry	Catalyst (amount of catalyst)	Condition	Time (min)	Yield^a^ (%)	References
1	Nano-ZnO (10 mol%)	EtOH/r. t	50	83	^46^
2	Nano-g-Alumina (10 mg)	EtOH/r. t	50	85	^44^
3	Nano-ZMS-5 (10 mg)	EtOH/r. t	55	90	^44^
4	Succinic acid (0.5 mmol)	EtOH: H_2_O/80 (°C)	150	92	^47^
5	PhB(OH)_2_ (10 mol%)	EtOH/reflux	5 h	82	^48^
6	PPh_3_ (20 mol%)	EtOH/reflux	120	81	^49^
7	NH_4_VO_3_(15 mg)	EtOH/reflux	20	93	This work

^a^Isolated yield.

**Figure 6:**
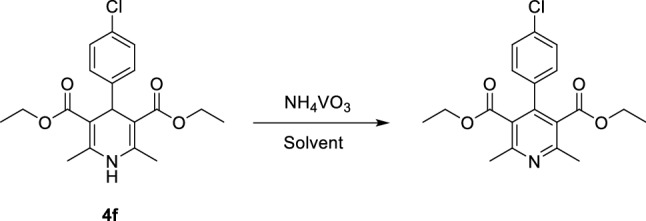
Oxidation of 1,4-DHPs by using NH_4_VO_3_.

We also extended our study to the oxidation of the synthesized 1,4-DHPs. Compound **4f.** (diethyl 4-(4-chloro phenyl)-2,6-dimethyl-1,4-dihydropyridine-3,5-dicarboxylate) was used as a model substrate to optimize the oxidation reaction conditions (Fig. 6).

As revealed in Table 5 (entries 1–8), the nature of the solvent is an important factor in the oxidation of 1,4-DHPs to the corresponding pyridines. The effect of the solvent in the oxidation reaction, in dichloromethane, ethanol, chloroform, H_2_O, acetonitrile, formic acid, and tetrahydrofuran was investigated; no oxidation occurred in these solvents. While by addition of acetic acid as the solvent to the reaction mixture, the yield of the desired product reached 96% under reflux conditions (entry 8, Table 5), this observation suggests that acetic acid is essential for the oxidation reaction. Additionally, the model substrate converts into the corresponding pyridine in acetic acid at room temperature (entry 9, Table 5). The model substrate was treated with 58.0–180.0 mg of NH_4_VO_3_ in the presence of acetic acid under reflux conditions (entries 10–16, Table 5). The satisfactory yield of the desired product can be obtained with 117.0 mg of NH_4_VO_3_ (entry 8, Table 5). The experiment was conducted in the oxygen, nitrogen, and air atmosphere (entries 8–11, Table 5), the oxidation reaction progressed in the nitrogen atmosphere the same as in normal reaction conditions using air or oxygen atmosphere.

**Table 5:** Optimization of reaction conditions in the oxidation of 1,4-DHPs.

Entry	Solvent	Time (min)	Amount of catalyst (mg)	Temperature	Yield^a^ (%)
1	Dichloromethane	1080	117	Reflux	0
2	Chloroform	1080	117	Reflux	0
3	Ethanol	1080	117	Reflux	0
4	Water	1080	117	Reflux	0
5	Acetonitrile	1080	117	Reflux	0
6	Formic acid	1080	117	Reflux	0
7	Tetrahydrofuran	1080	117	Reflux	0
8	Acetic acid	10	117	Reflux	96
9	Acetic acid	120	117	r. t	85
10	Acetic acid	10	117	Reflux	96^b^
11	Acetic acid	10	117	Reflux	96^c^
12	Acetic acid	60	58	Reflux	73
13	Acetic acid	60	88	Reflux	85
14	Acetic acid	60	116	Reflux	90
15	Acetic acid	10	120	Reflux	96
16	Acetic acid	10	180	Reflux	96

Reaction conditions: 1,4-DHPs (1.0 mmol), solvent (3.0 mL). ^a^Isolated yields. ^b^Under N_2_ atmosphere. ^c^Under O_2_ atmosphere.

Under the optimized reaction conditions, the catalytic performance of NH_4_VO_3_ was further evaluated for the oxidation reaction of various 1,4-DHPs containing electron-withdrawing and donating substituents (Table 7). The Hantzsch 1,4-DHPs including a variety of substituents were converted to the corresponding pyridines in excellent yield (Table 7). Based on the results for the oxidation of 1,4-DHPs by other catalysts reported previously (Table 6), the NH_4_VO_3_ can act as a highly efficient heterogeneous catalyst in oxidation reaction through a facile method (Table 7).
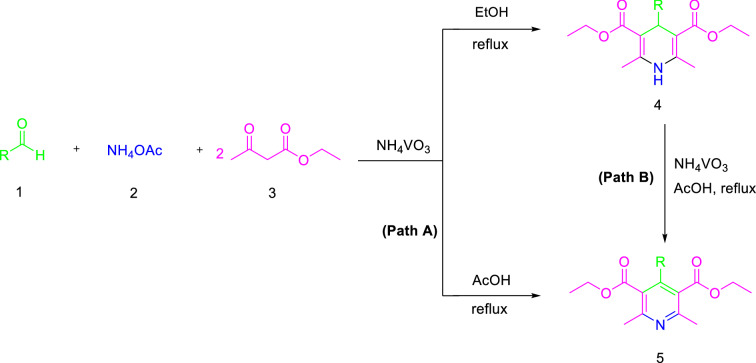

**Table 6:** Comparison of the results for the oxidation of 1,4-DHP (4f.) using other catalysts.

Entry	Catalyst	Condition	Time	Yield^a^ (%)	References
1	CuBr_2_ (3 mmol)	CH_3_COOCH_2_CH_3_/CHCl_3_/reflux	2 h	81	^50^
2	TBA-eosinY/ K_2_CO_3_ (1 mol %)	Methanol/water/LED irradiation/Air	12 h	85	^51^
3	H_2_O_2_/V_2_O_5_(5 mol %)	CH_3_CN/r. t	1 h	95	^52^
4	PhCH_2_Ph_3_PHSO_5_/BiCl_3_(1 eq/3 eq)	CH_3_CN/r. t	1/40 h	81	^53^
5	NHPI/Co(OAc)_2_·4H_2_O (20 mol %/0.5 mol %)	CH_3_CN/Air/reflux	4 h	98	^54^
6	NH_4_VO_3_(117 mg)	AcOH/reflux	10 min	98	This work

^a^Isolated yield.

**Table 7:** Synthesis of pyridine derivatives and 1,4-DHPs in the presence of NH_4_VO_3_ as the catalyst

Entry	Aldehyde (**1**)	Product (**5**) Path **A**^a^		Product (**5**) Path **B**^b^		Mp (°C) ^ref^ Product (**5**)	Product (**4**)^c^		Mp (°C) ^ref^ Product (**4**)
							Time (min)	Yield^d^ (%)	
		Time (min)	Yield^d^ (%)	Time (min)	Yield^d^ (%)				
1	Formaldehyde	5	99	5	98	69–70^55^	20	65	165–168^56^
	
2	PhCHO	10	99	15	96	59–61^53^	15	80	151–153^54^
	
3	4-(Me)C_6_H_4_CHO	10	95	15	97	71–73^29^	20	87	133–136^54^
	
4	4-(OMe)C_6_H_4_CHO	10	100	25	96	57–58^44^	10	90	163–165^54^
	
5	4-(Br)C_6_H_4_CHO	10	99	10	98	51–53^29^	15	95	160–162^54^
	
6	4-(Cl)C_6_H_4_CHO	10	96	10	97	71–72^29^	20	93	144–147^57^
	
7	4-(F)C_6_H_4_CHO	10	99	15	98	88–89^29^	20	90	153–156^58^
	
8	4-(OH)C_6_H_4_CHO	15	99	20	95	171–174^44^	60	85	227–230^54^
	
9	3-(OH)C_6_H_4_CHO	15	99	10	97	150–153^59^	45	88	187–189^54^
	
10	3-(NO_2_)C_6_H_4_CHO	10	98	30	95	60–61^53^	40	91	163–166^54^
	
11	4-(CN)C_6_H_4_CHO	20	99	10	99	100–102^29^	15	96	194–196^60^
	
12	Furan-2-carbaldehyde	10	80	10	98	Oil	16	98	161–163^61^
	
13	Thiophen-2-carbaldehyde	40	75	10	98	37–39^44^	15	97	168–170^55^
	
14	Cinnamaldehyde	10	65	25	80	161–162^44^	20	98	148–150^54^
	
15	Terephthalaldehyde	180	83	180	90	211–213^62^	30	97	279–283^63^
	

^a^Reaction conditions: aldehyde (1.0 mmol), ethyl acetoacetate (2.0 mmol), ammonium acetate (2.0 mmol), AcOH (3.0 mL), NH_4_VO_3_ (117.0 mg), under air condition. ^b^Reaction conditions: 1,4-dihydropyridines (1.0 mmol), AcOH (3.0 mL), NH_4_VO_3_ (117.0 mg), under air condition. ^c^Reaction conditions: aldehyde (1.0 mmol), ethyl acetoacetate (2.0 mmol), ammonium acetate (2.0 mmol), EtOH (3.0 mL), NH_4_VO_3_ (15.0 mg). ^d^Isolated yields. ^e^Reaction conditions: aldehyde (1.0 mmol), ethyl acetoacetate (4.0 mmol), ammonium acetate (4.0 mmol), AcOH (3.0 mL), NH_4_VO_3_ (150.0 mg).

## Conclusion

In conclusion, a novel and convenient approach for the one-pot synthesis of pyridine derivatives through the one-pot pseudo four-component reaction, and oxidation of 1,4-DHPs by using NH_4_VO_3_ as the catalyst has been developed. NH_4_VO_3_ is an efficient, commercially available, inexpensive, and eco-friendly catalyst for these reactions. These methods involve several remarkable advantages, such as simple procedure, mild reaction conditions, short reaction times, high yields, and ease of product isolation.

## Supplementary Information

Supplementary Information.

## Data Availability

All data generated or analyzed during this study are included in this published article and its supplementary information file. The data is also available through request from corresponding author.
