# Supplementary material for: Ammonium metavanadate (NH4VO3): a highly efficient and eco-friendly catalyst for one-pot synthesis of pyridines and 1,4-dihydropyridines
Source: Sci Rep. 2022 Aug 11;12:13687. doi: 10.1038/s41598-022-17378-7 (PMC9372032; doi:10.1038/s41598-022-17378-7)
Supplement: Supplementary file 1 — Supplementary Information. [file 41598_2022_17378_MOESM1_ESM.docx]

**Supplementary Information**

**Ammonium metavanadate (NH_4_VO_3_): A highly efficient and eco-friendly catalyst for one-pot synthesis of pyridines and 1,4-dihydropyridines**

Jamal Rahimi^a^, Maryam Niksefat^a^, Marzieh Heidari^b^, Mehdi Naderi^a^, Hadis Abbasi^a^, Mohammad Tajik Ijdani^a^, Ali Maleki^a^*

*^a^Catalysts and Organic Synthesis Research Laboratory, Department of Chemistry, Iran University of Science and Technology, Tehran 16846-13114, Iran.*

*^b^Department of Chemistry, Rutgers University, 73 Warren Street, Newark, NJ, 07102, USA.*

**Corresponding author e-mail:* [*maleki@iust.ac.ir*](mailto:maleki@iust.ac.ir)*; Fax: +98-21-73021584; Tel: +98-21-77240540-50*

| **Table of contents** |
| --- |
| Subject Page |
| Figure S1. FT-IR spectrum of the product **5d** .......……………………………………..…………………...S3 |
| Figure S2-S3. ^1^H NMR spectrum of the product **5d** ..……………………………………….…..……...S4-S5 |
| Figure S4. FT-IR spectrum of the product **5e** .….………………………………………………..….……...S6  Figure S5-S7. ^1^H NMR spectrum of the product **5e** .……………………………………………………S7-S9 |
| Figure S8. FT-IR spectrum of the product **5f** ……….……………………………………………..……....S10  Figure S9-S11. ^1^H NMR spectrum of the product **5f** ..………………………………………...….…..S11-S13  Figure S12. FT-IR spectrum of the product **5m** ……..…………………….….……………..………….…S14  Figure S13-S15. ^1^H NMR spectrum of the product **5m** ..……………………………………….…....S15-S17  Figure S16. FT-IR spectrum of the product **4d** ...….………………………………………………..….….S18  Figure S17-S19. ^1^H NMR spectrum of the product **4d** .....……….……….….……………………....S19-S21  Figure S20. FT-IR spectrum of the product **4e** …….…………………………………………….…..……S22  Figure S21-S23. ^1^H NMR spectrum of the product **4e**..….….…...………………………….…..……S23-S25  Figure S24. FT-IR spectrum of the product **4f** …………………………………………………..….….….S26 |
| Figure S25. ^1^H NMR spectrum of the product **4f** …....……………………………...…..………………...S27 |
| Figure S26. ^13^C NMR spectrum of the product **4f** ………………………………….………….…..……...S28 |
| Figure S27. FT-IR spectrum of the product **4j**  ……………………………………………..…..….……..S29  Figure S28. ^1^H NMR spectrum of the product **4j** …..……………………………………..………………S30 |
| Figure S29. ^13^C NMR spectrum of the product **4j** ……….……………………………………..…..……..S31  Figure S30. FT-IR spectrum of the product **4f** ..……………………………………………..……...……..S32  Figure S31-S33. ^1^H NMR spectrum of the product **4f** ……………….….……………..………….…S33-S35 |


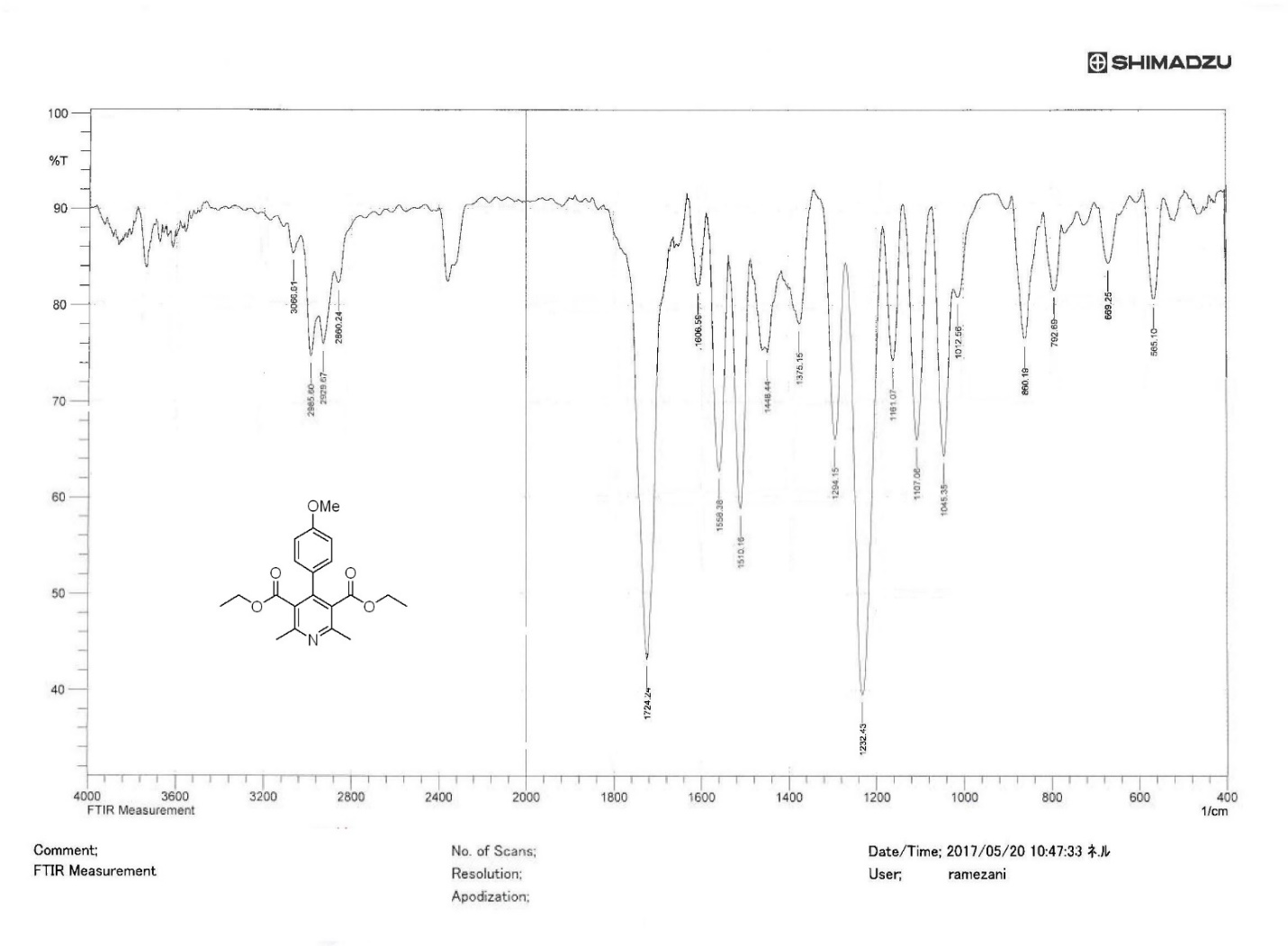
**Figure S1.** FT-IR spectrum of the product **5d.**


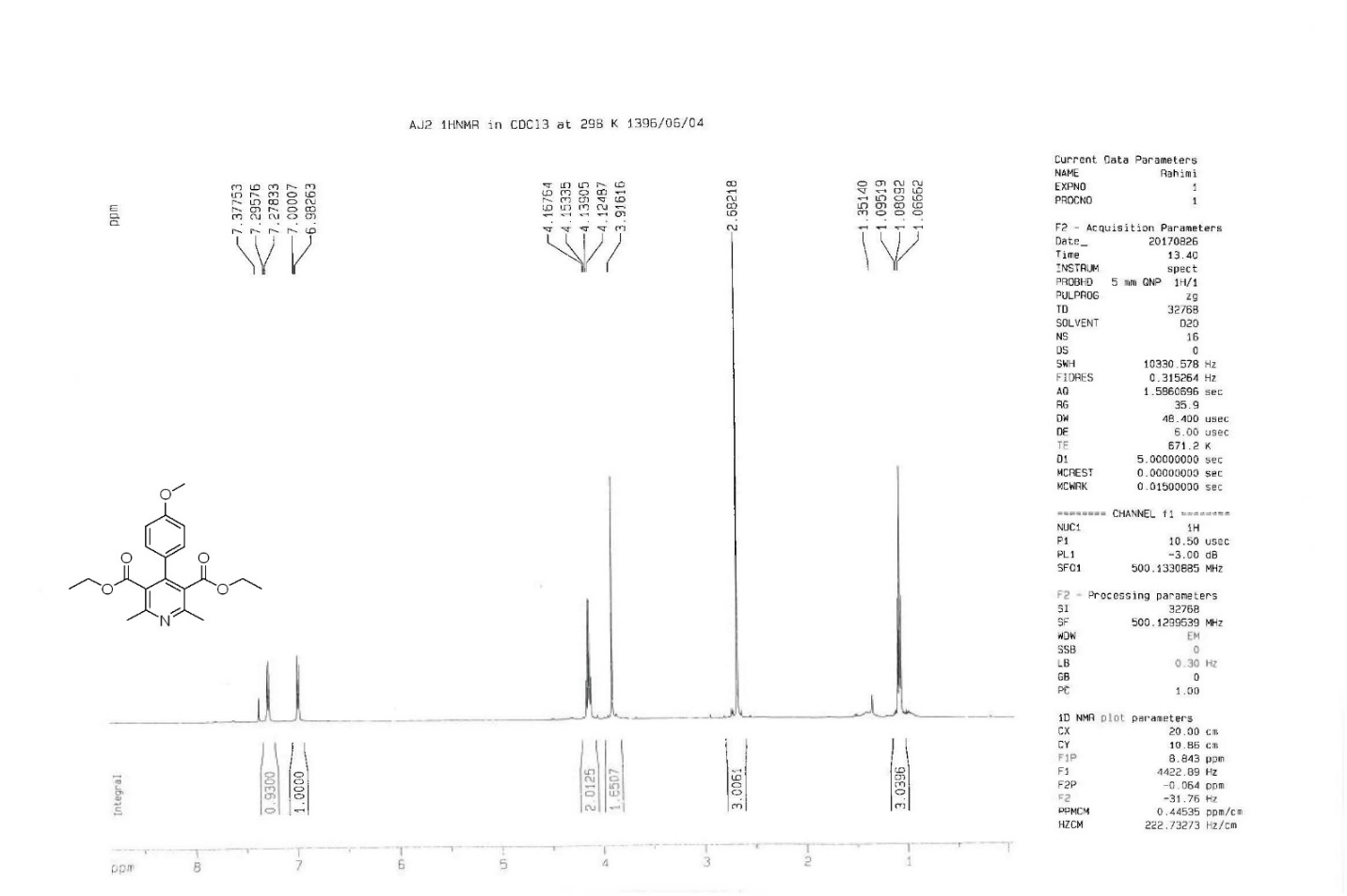


**Figure S2.** ^1^H NMR spectrum of the product **5d.**


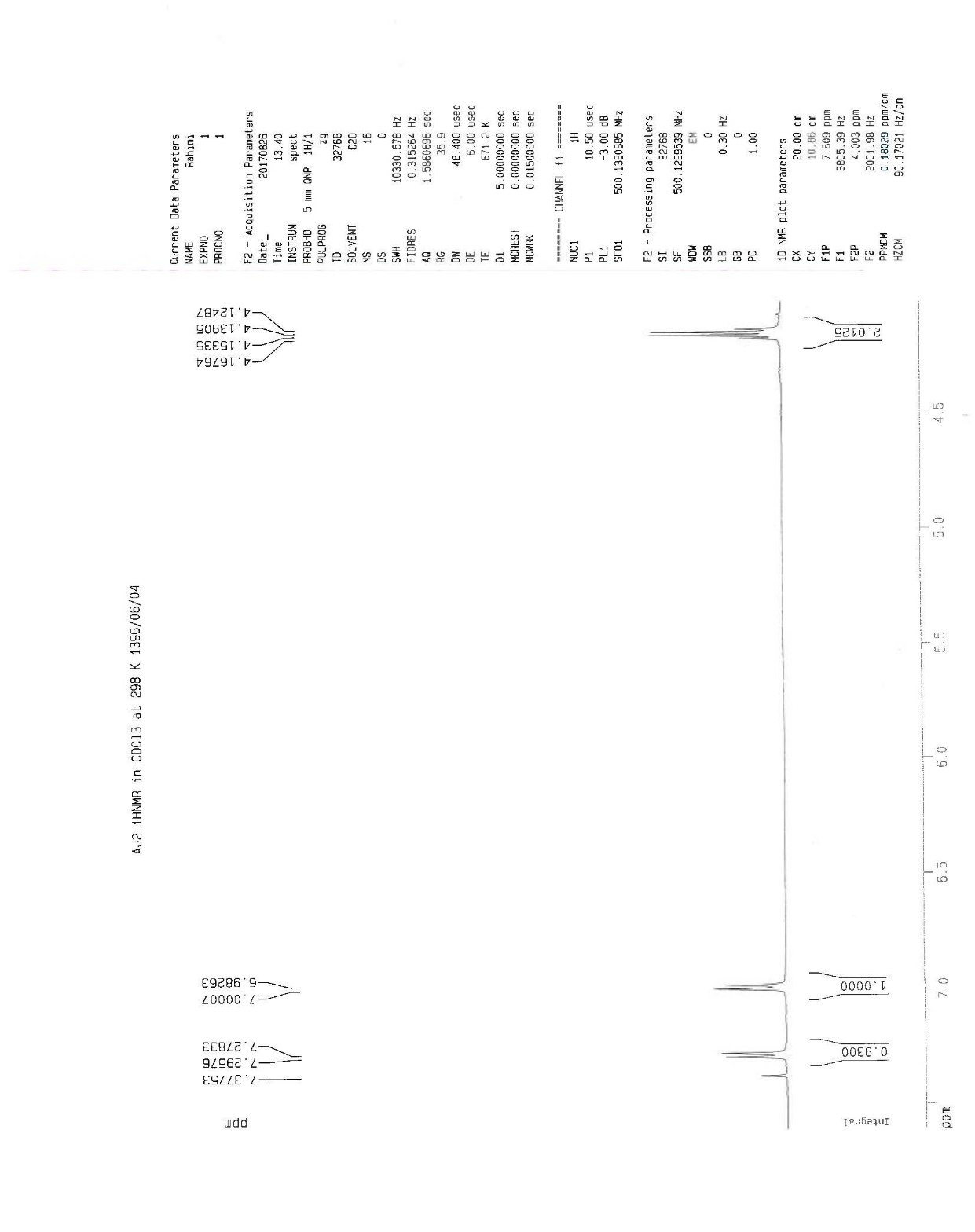


**Figure S3.** ^1^H NMR spectrum of the product **5d.**


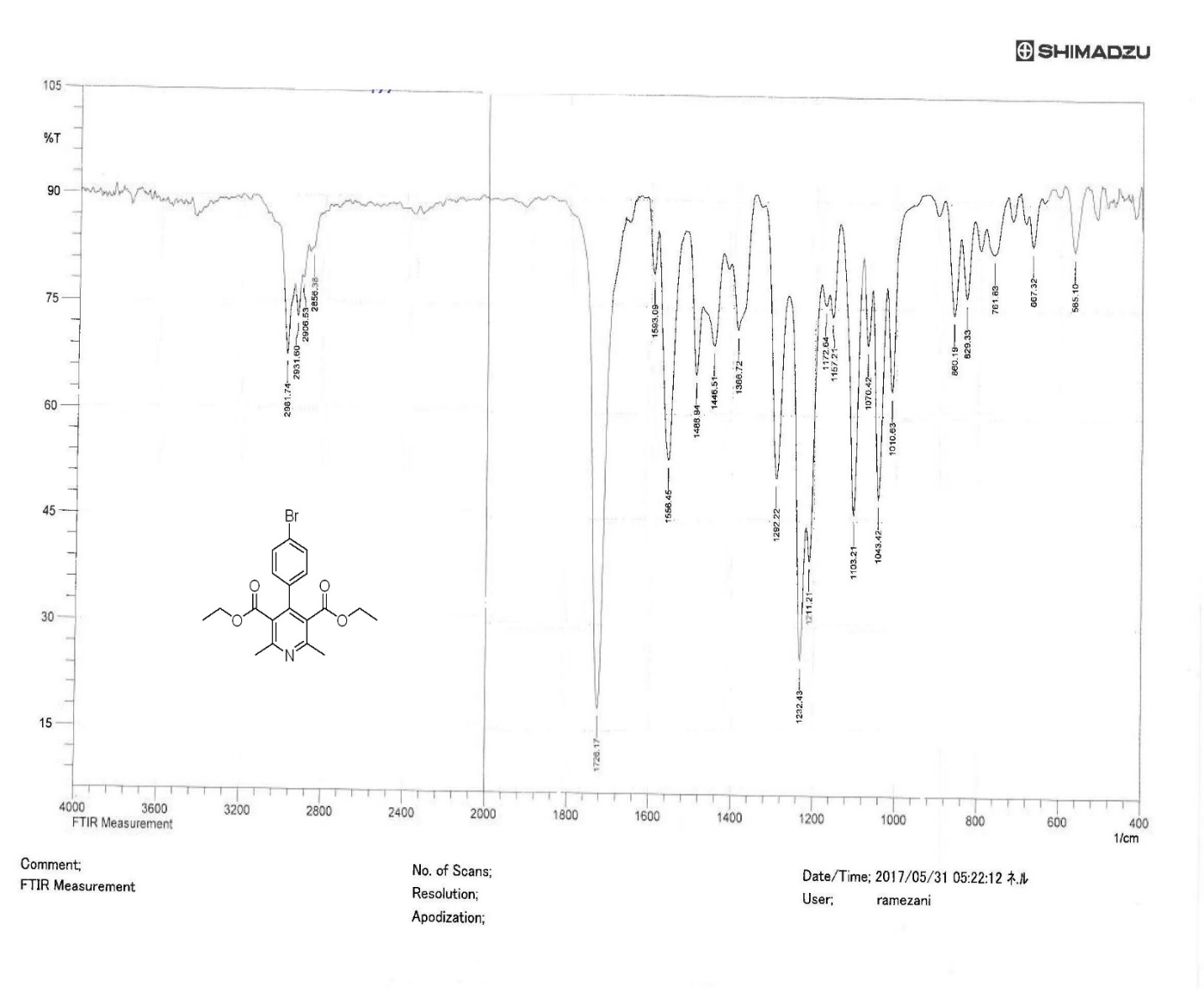


**Figure S4.** FT-IR spectrum of the product **5e.**


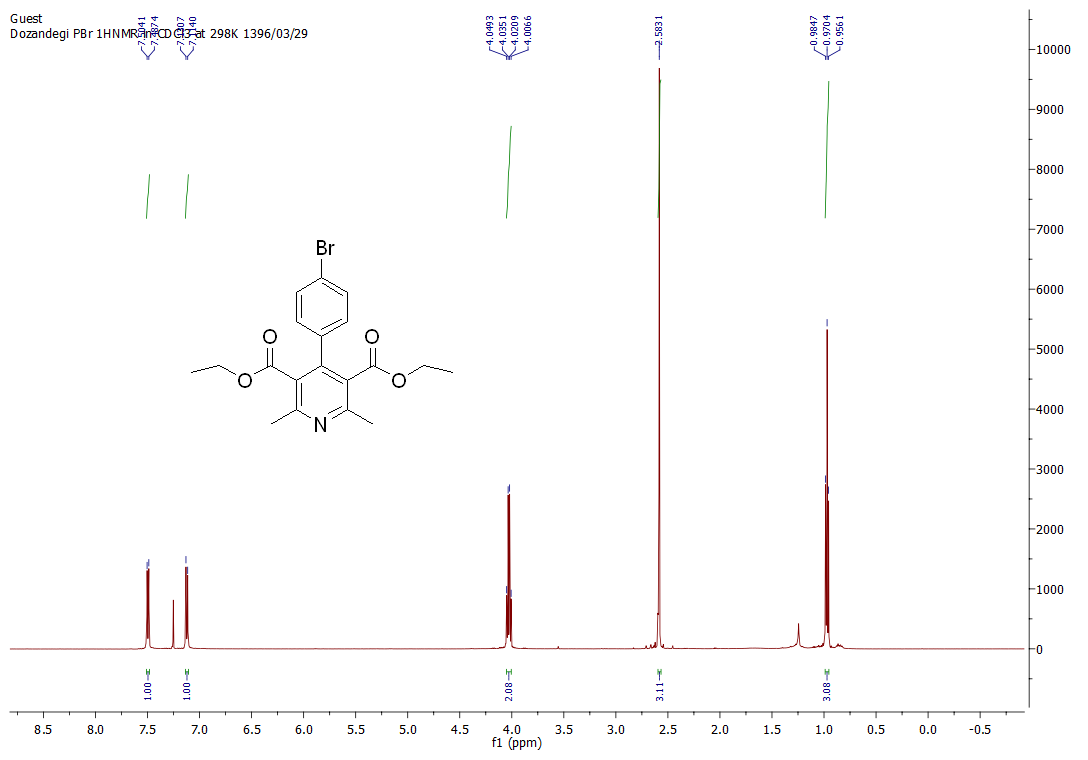


**Figure S5.** ^1^H NMR spectrum of the product **5e.**


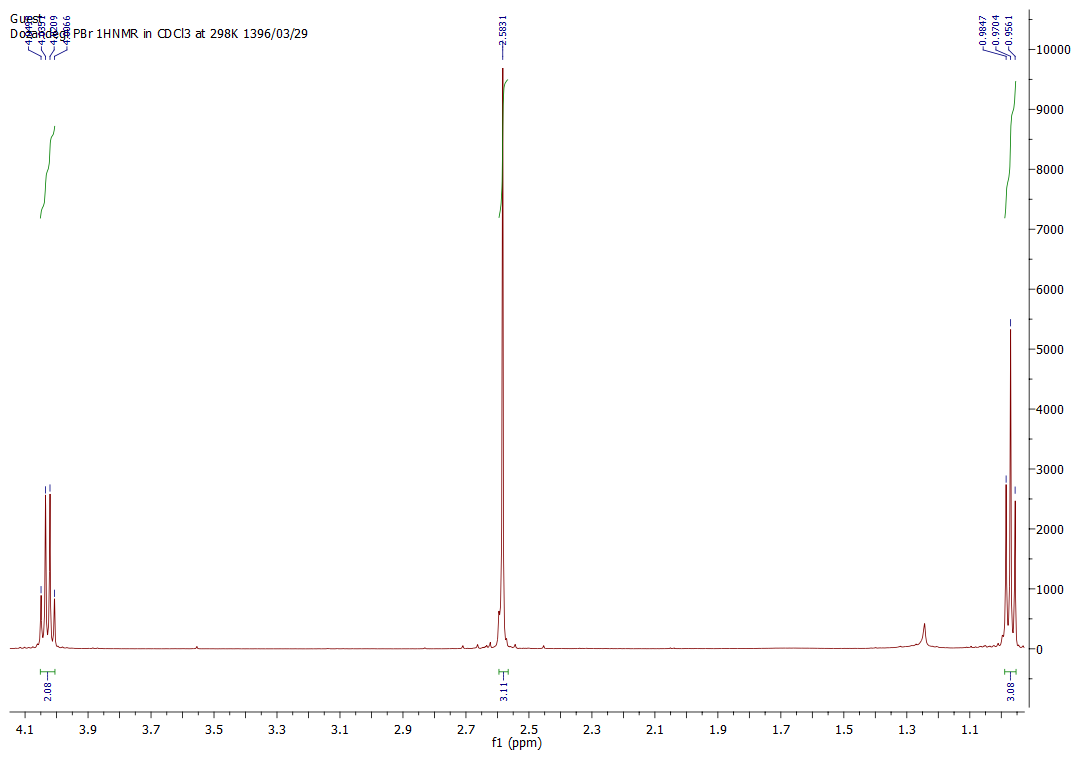


**Figure S6.** ^1^H NMR spectrum of the product **5e.**


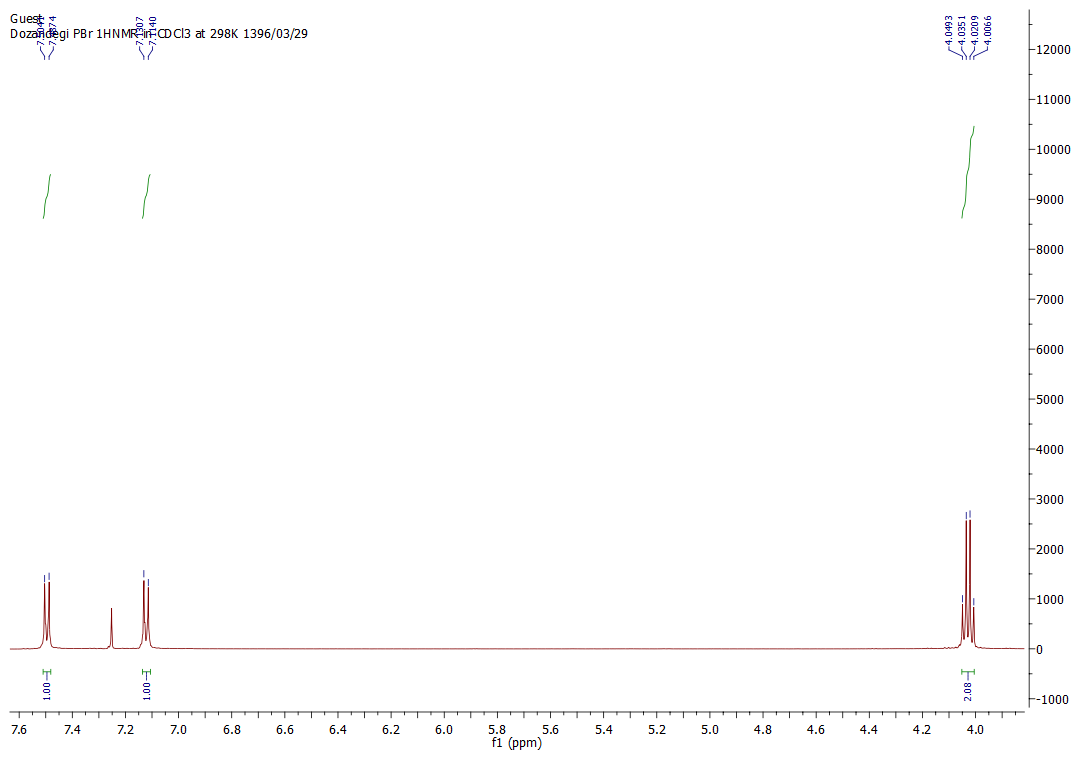


**Figure S7.** ^1^H NMR spectrum of the product **5e.**


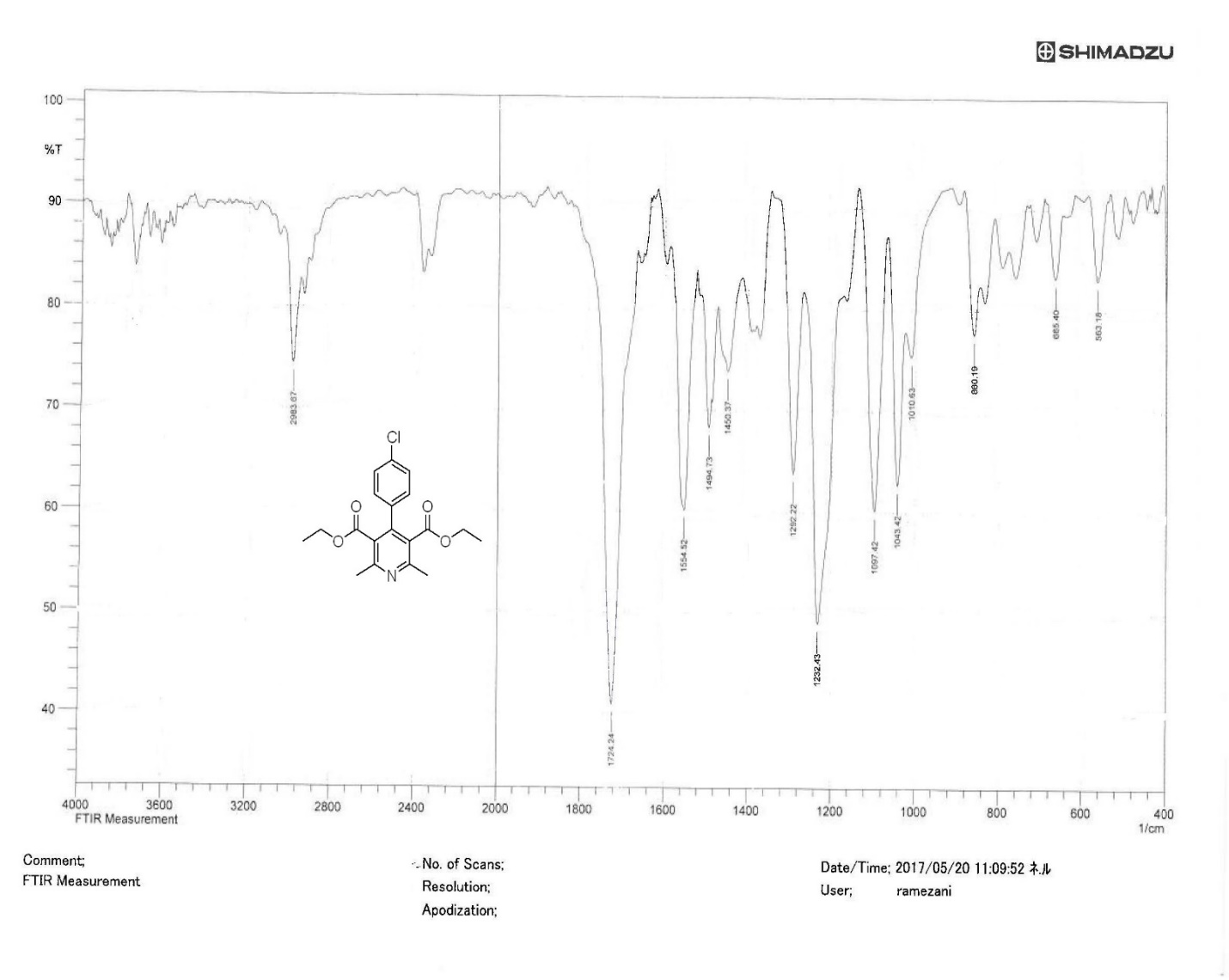


**Figure S8.** FT-IR spectrum of the product **5f.**


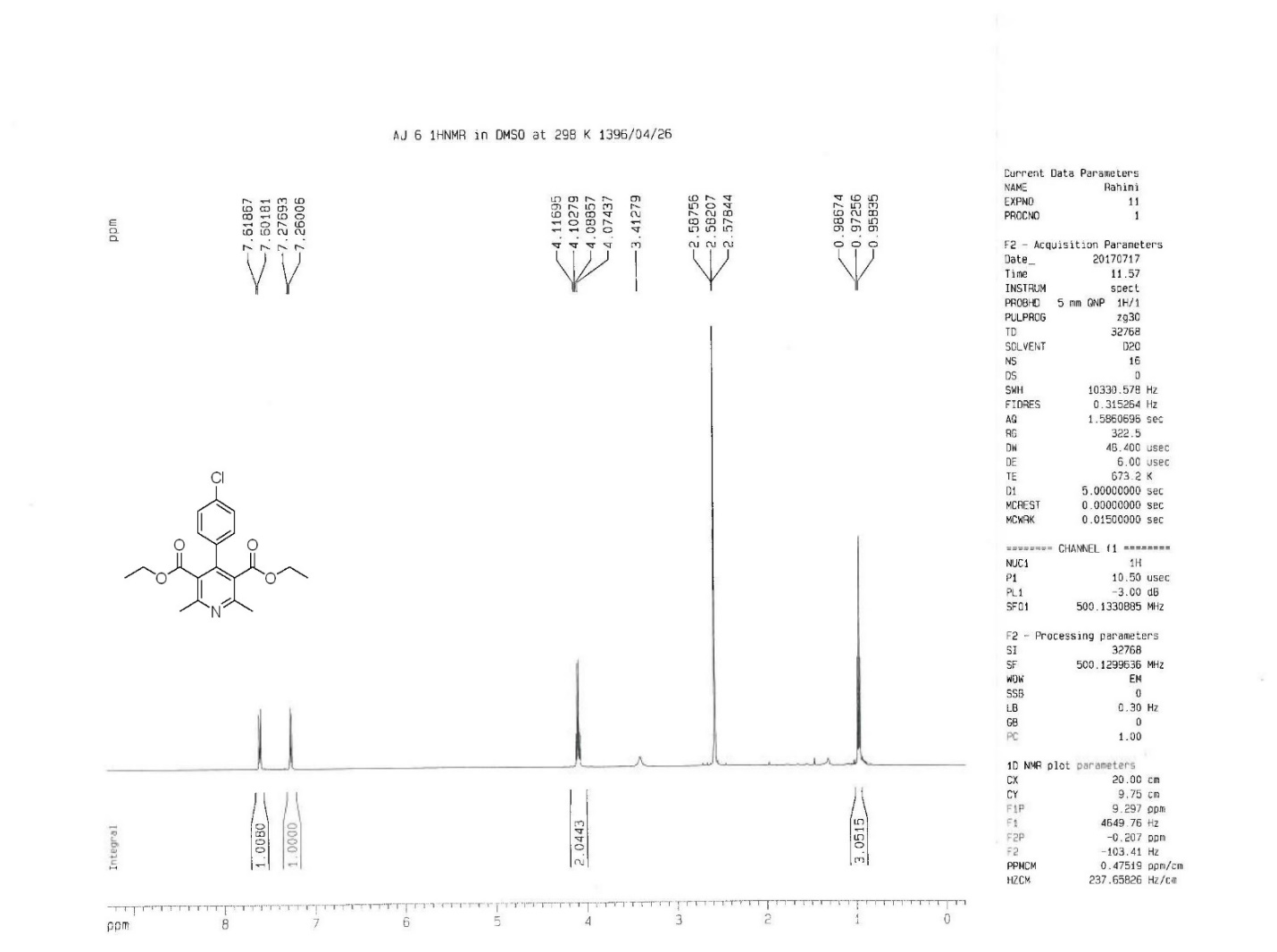


**Figure S9.** ^1^H NMR spectrum of the product **5f.**


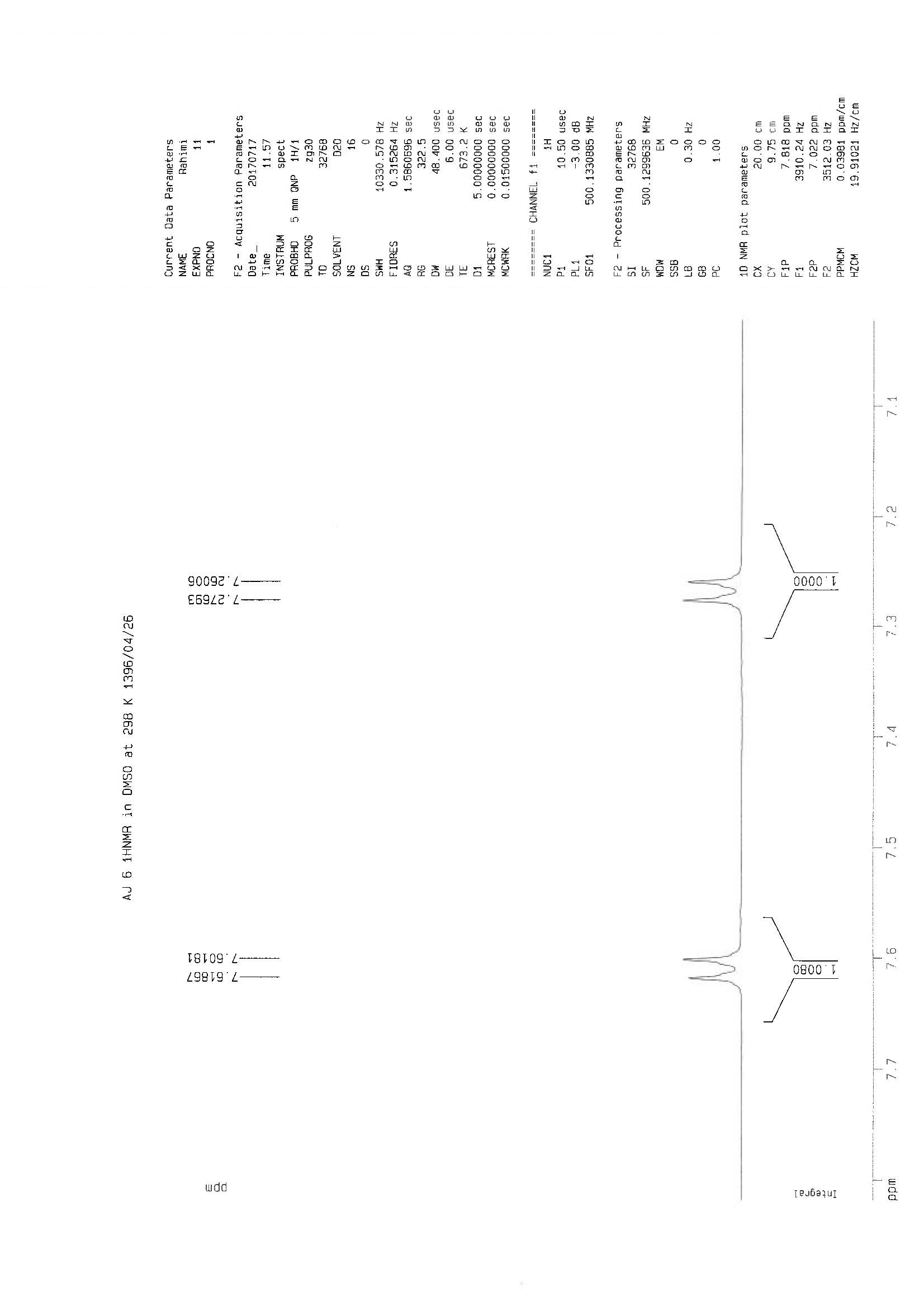


**Figure S10.** ^1^H NMR spectrum of the product **5f.**


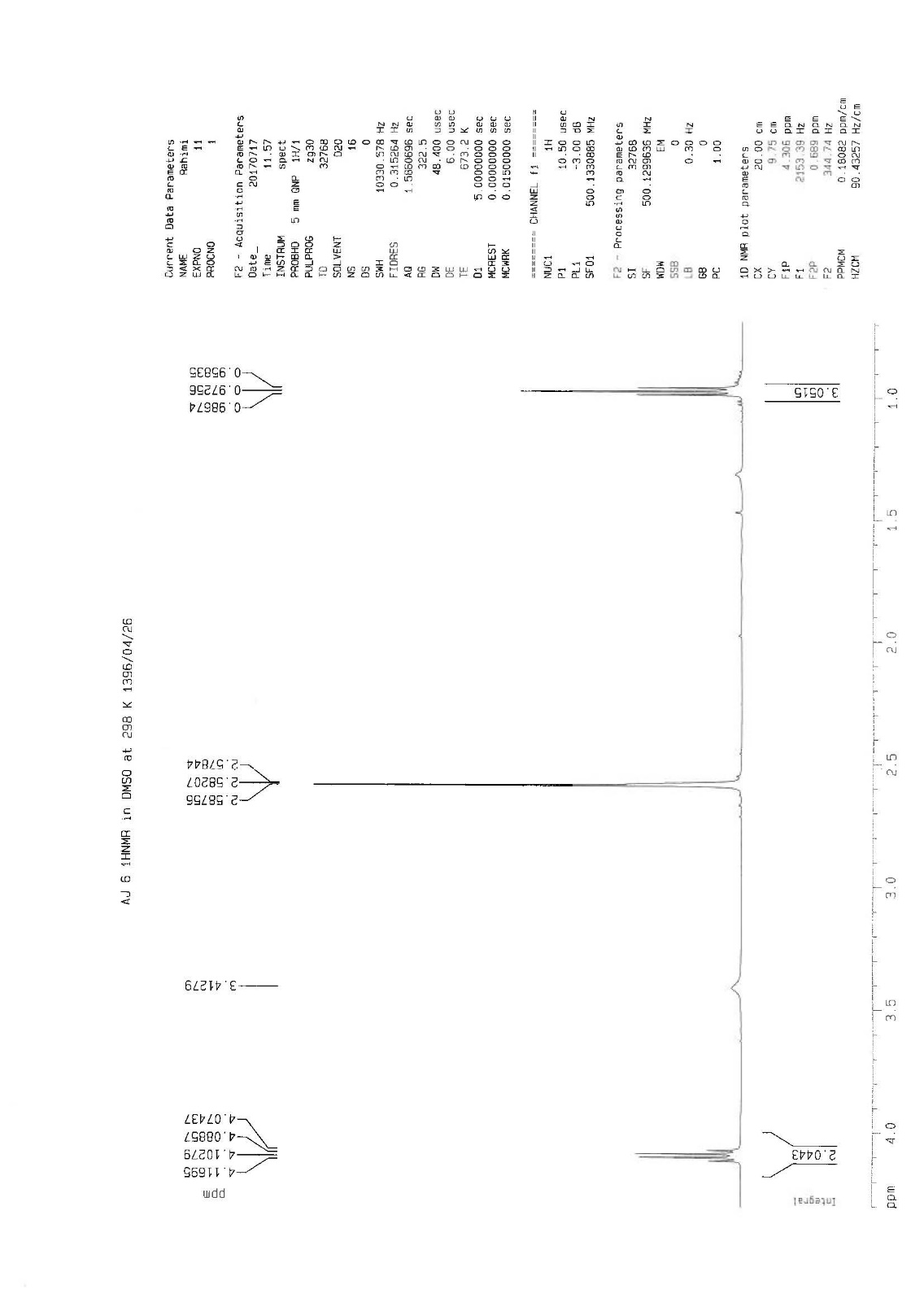


**Figure S11.** ^1^H NMR spectrum of the product **5f.**


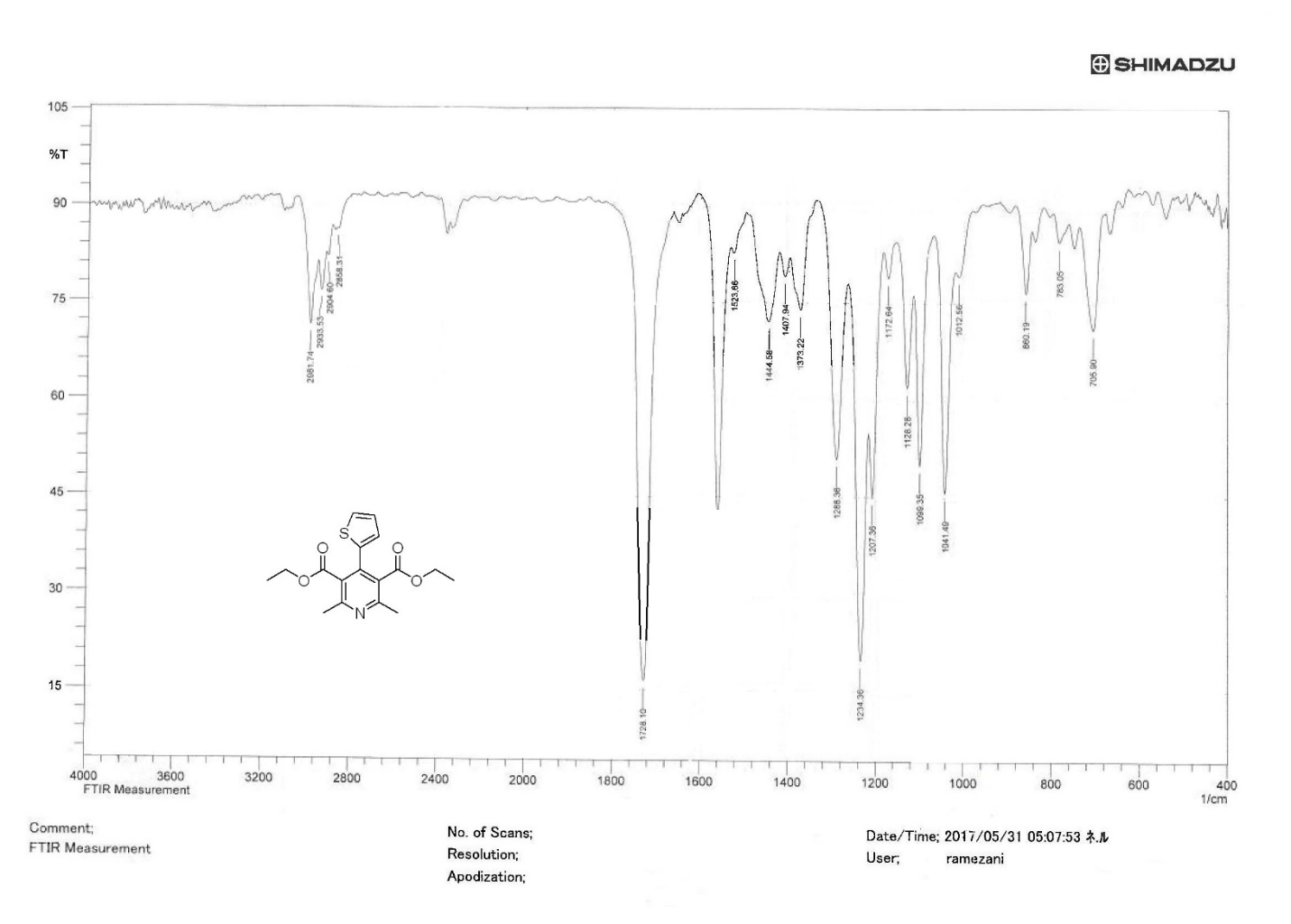
 **Figure S12.** FT-IR spectrum of the product **5m.**


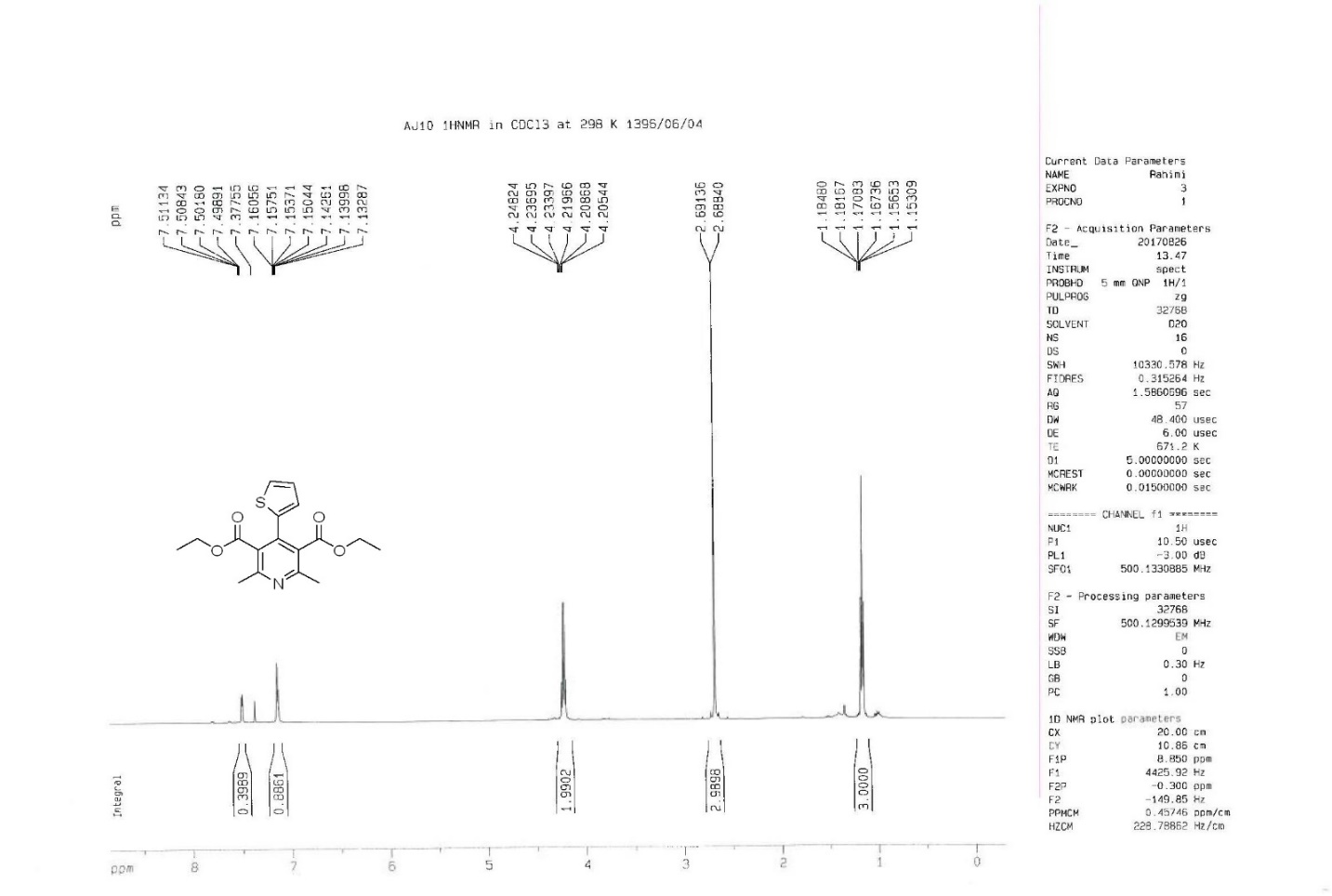


**Figure S13.** ^1^H NMR spectrum of the product **5m.**


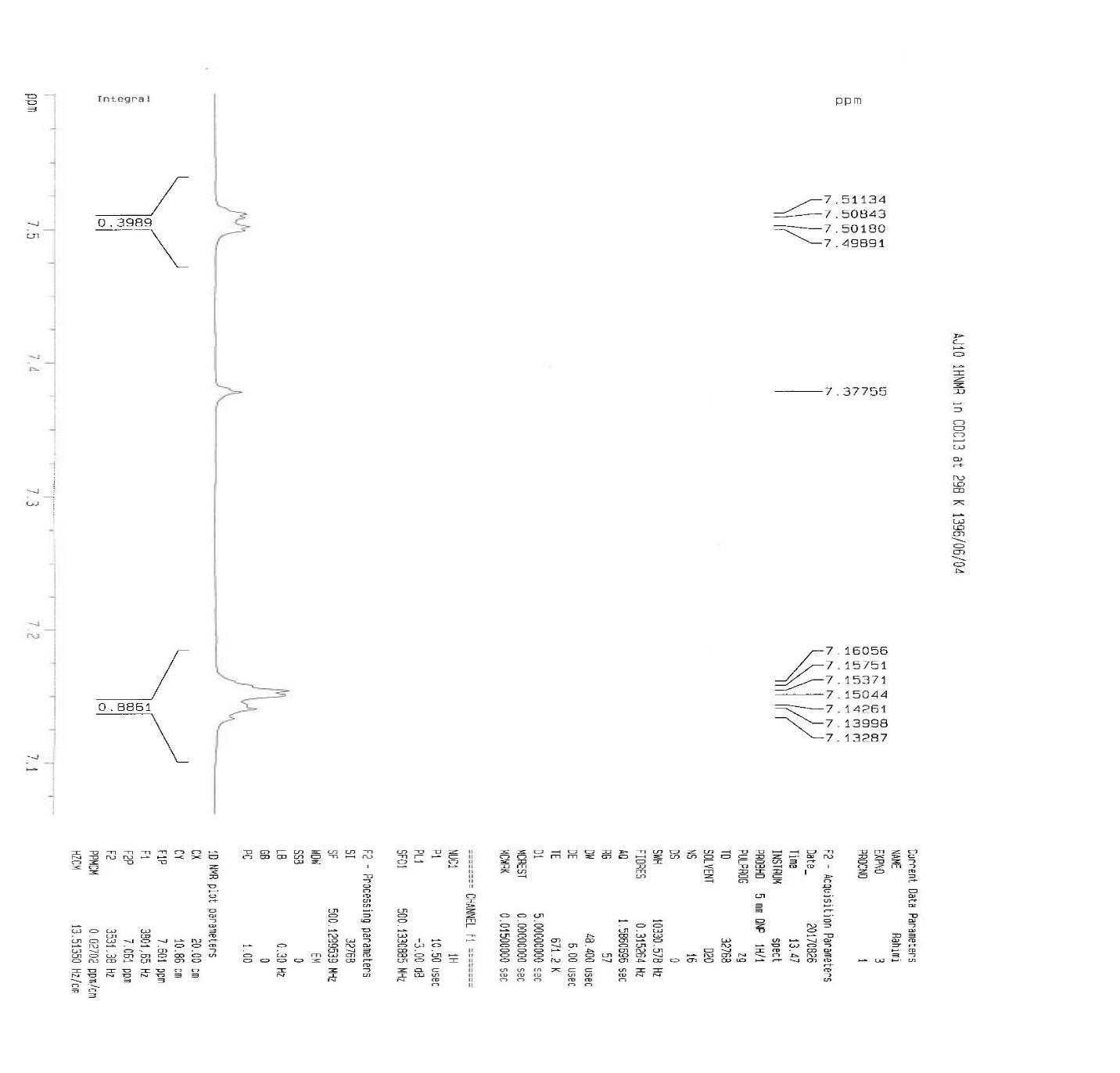


**Figure S14.** ^1^H NMR spectrum of the product **5m.**


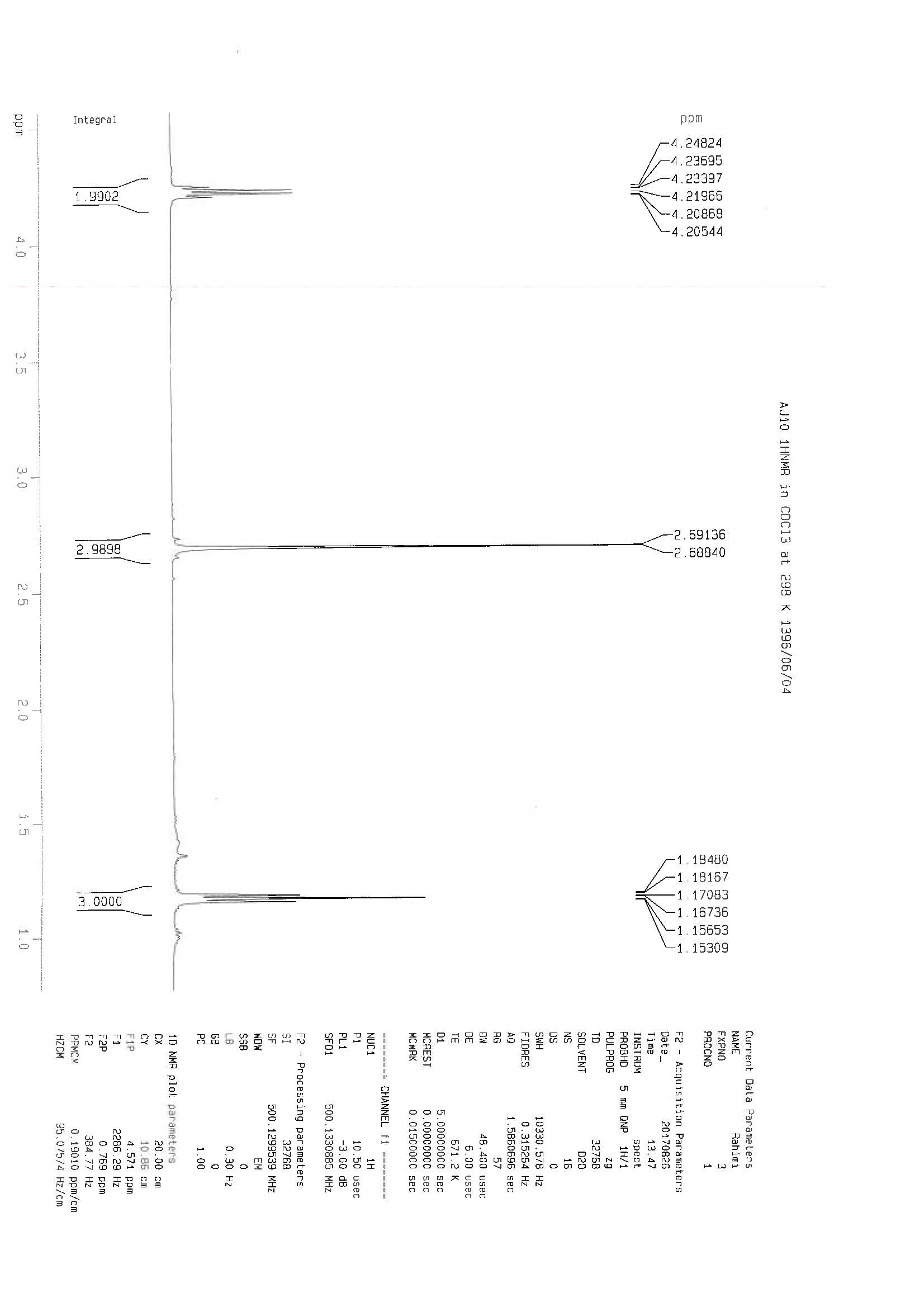


**Figure S15.** ^1^H NMR spectrum of the product **5m.**


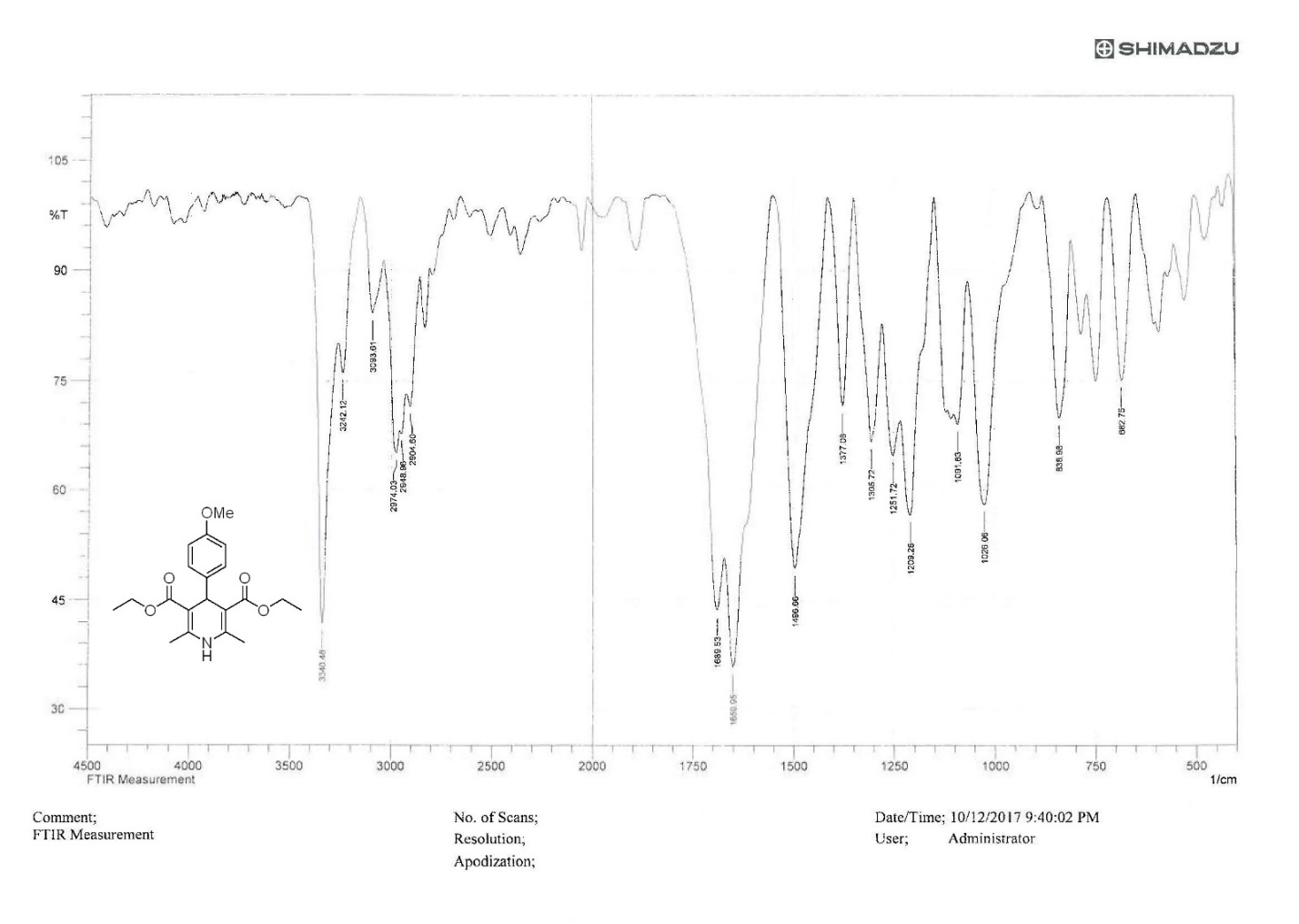


**Figure S16.** FT-IR spectrum of the product **4d.**


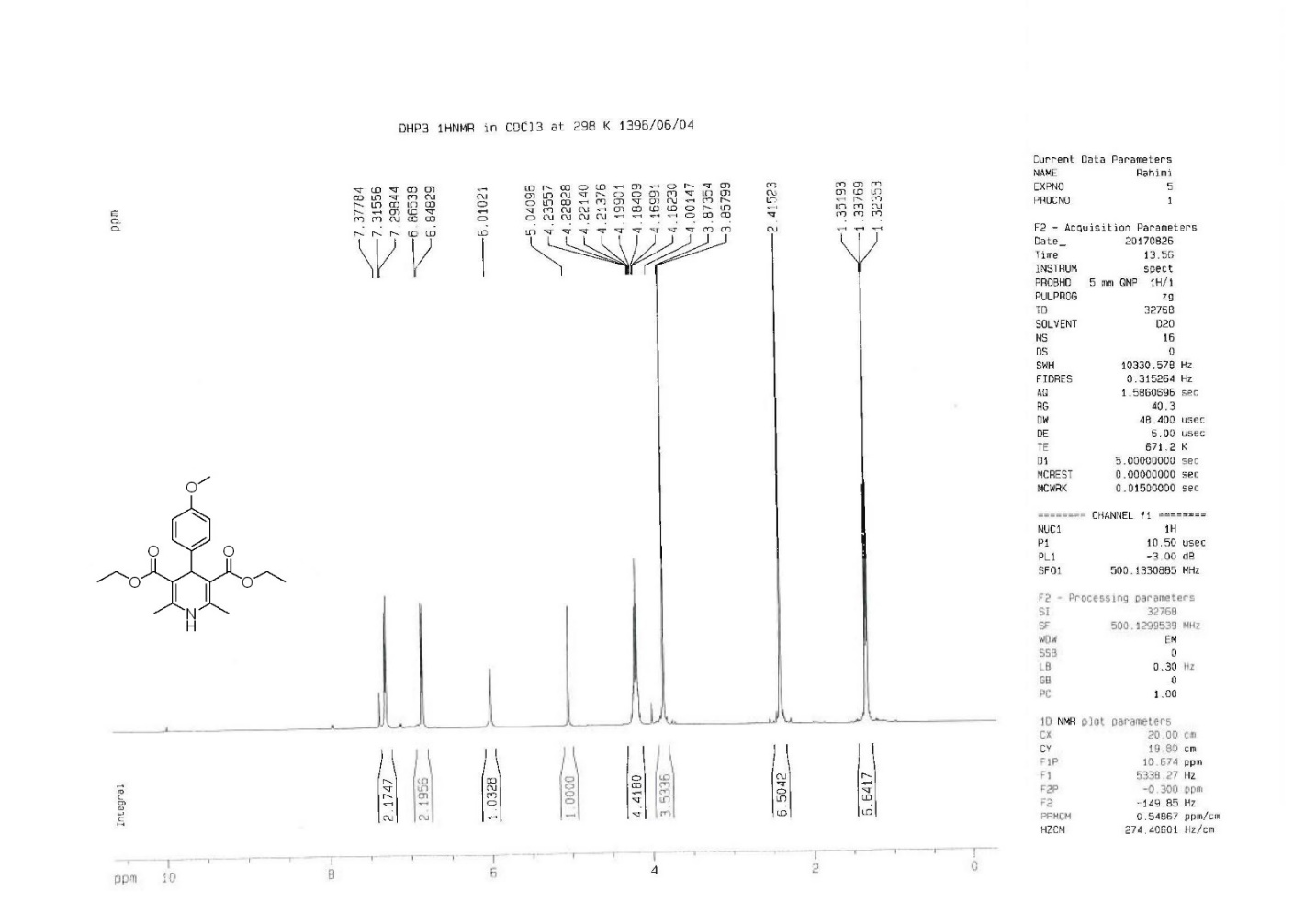


**Figure S17.** ^1^H NMR spectrum of the product **4d.**


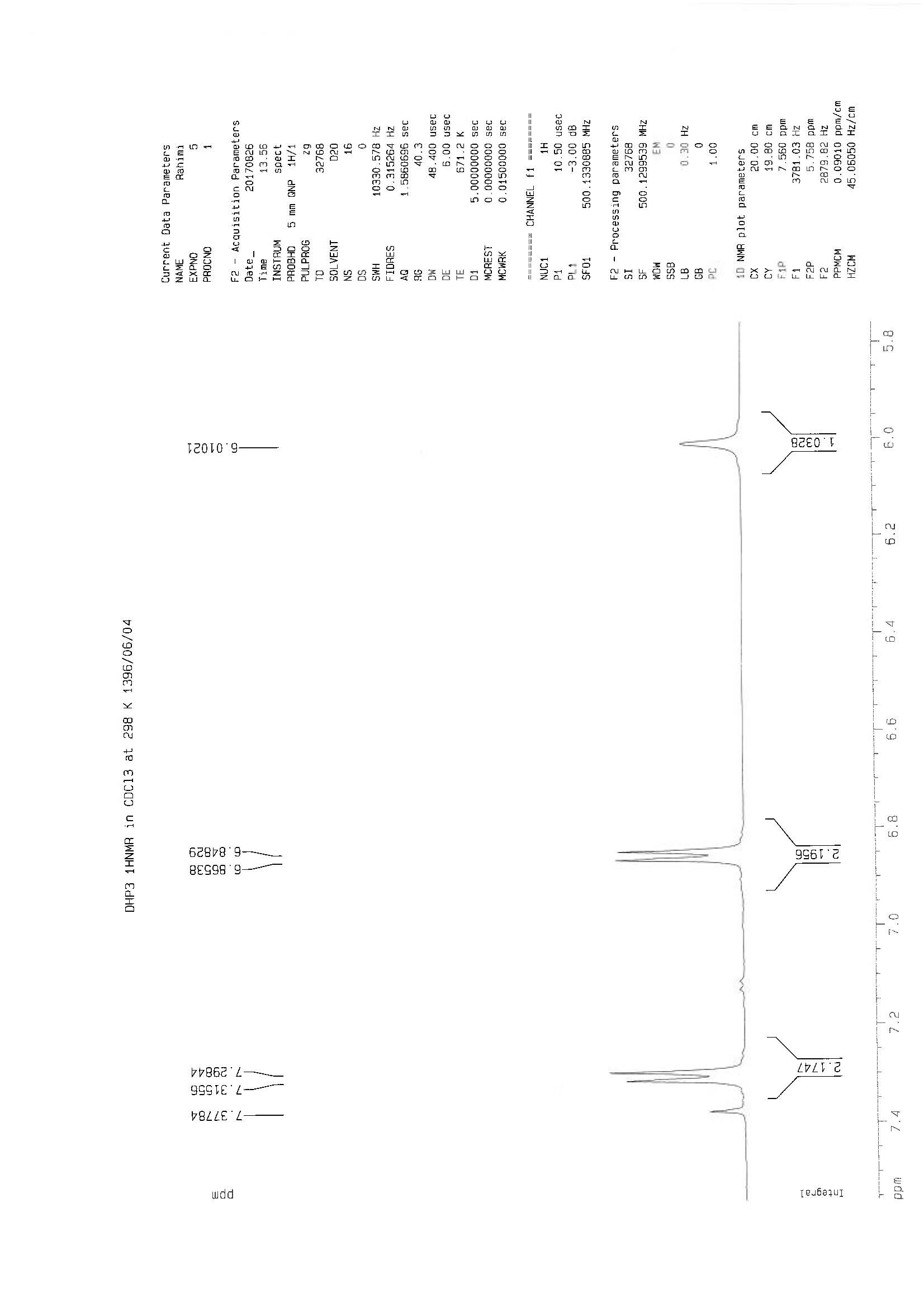


**Figure S18.** ^1^H NMR spectrum of the product **4d.**


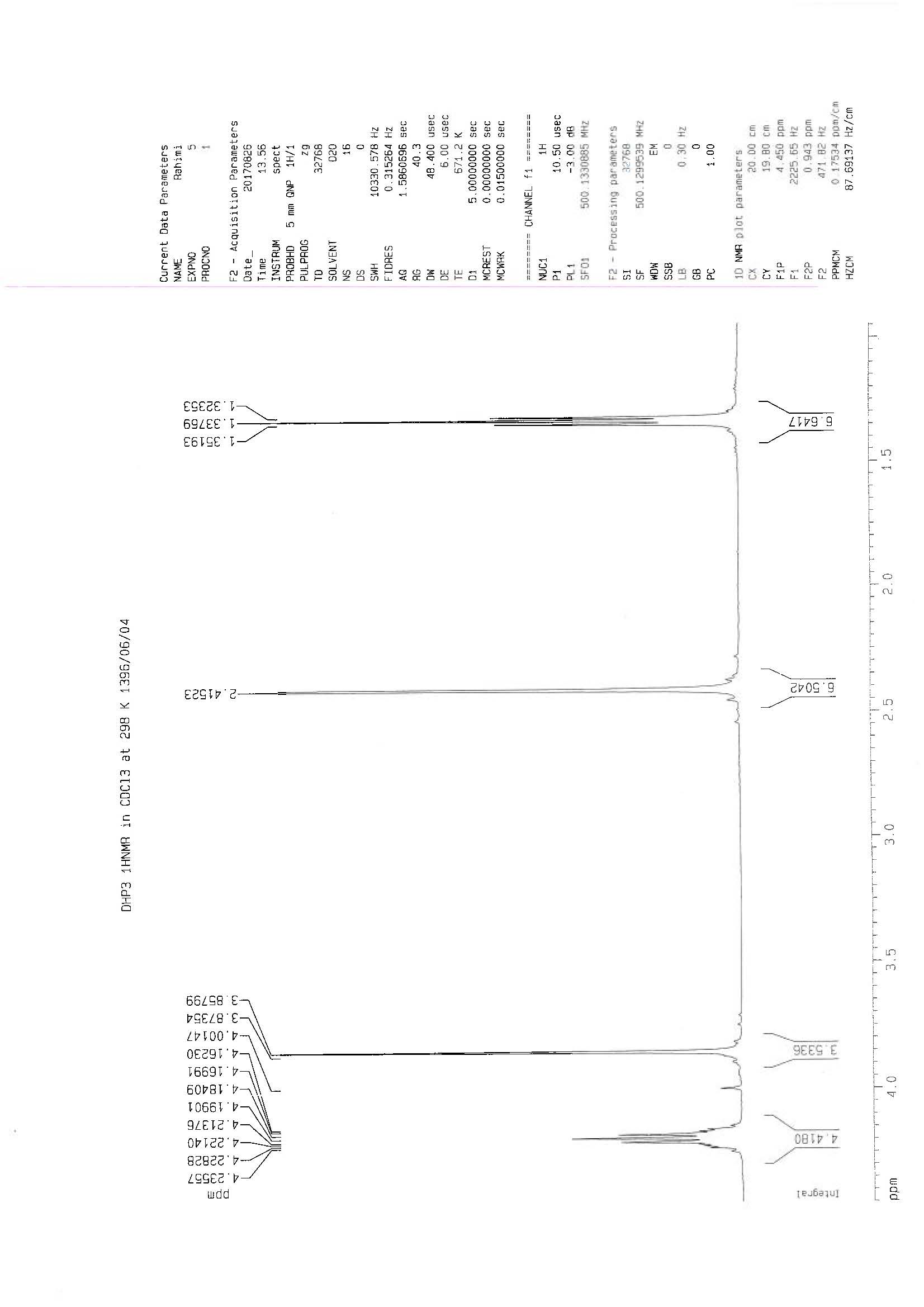


**Figure S19.** ^1^H NMR spectrum of the product **4d.**


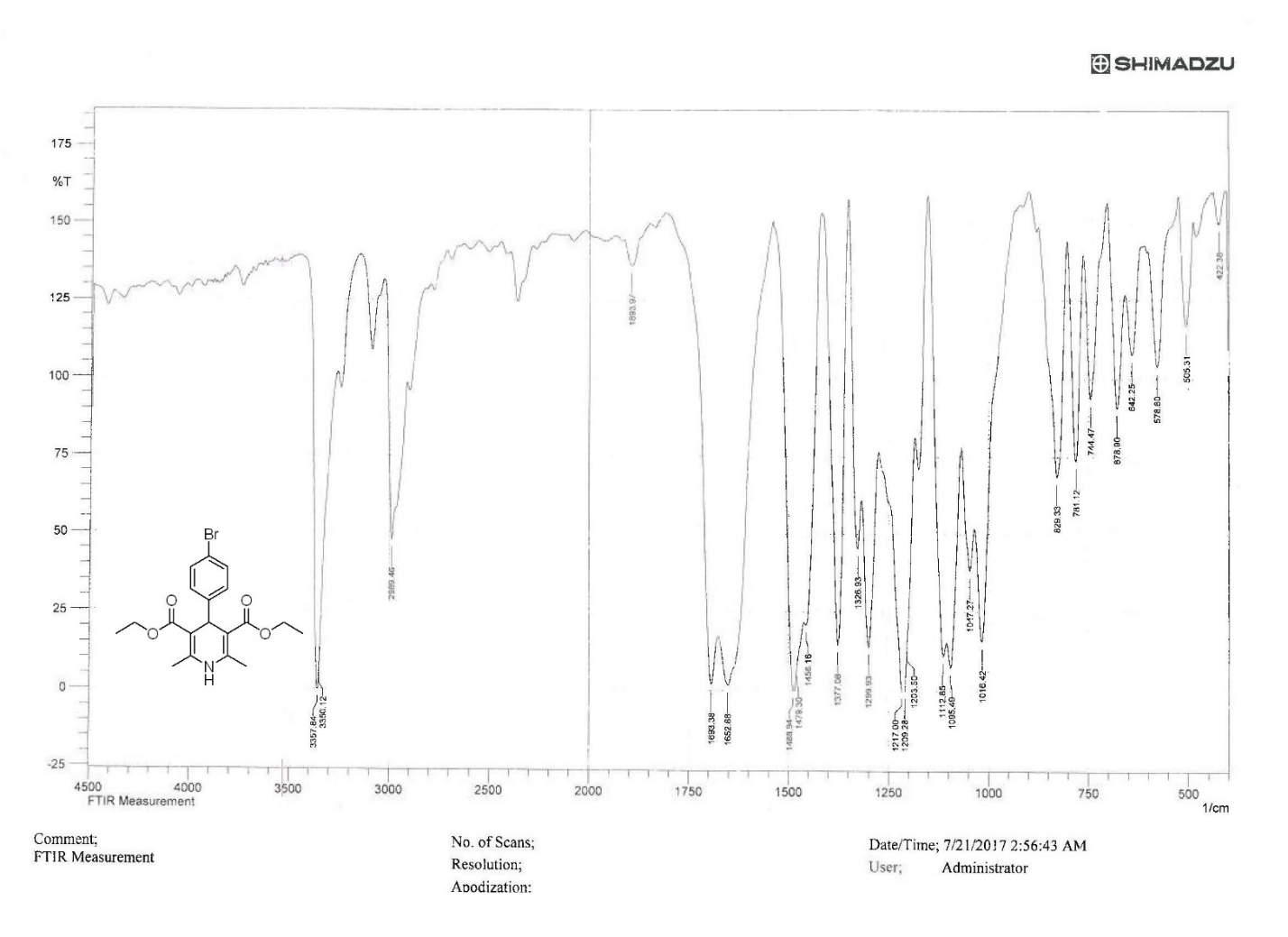


**Figure S20.** FT-IR spectrum of the product **4e.**


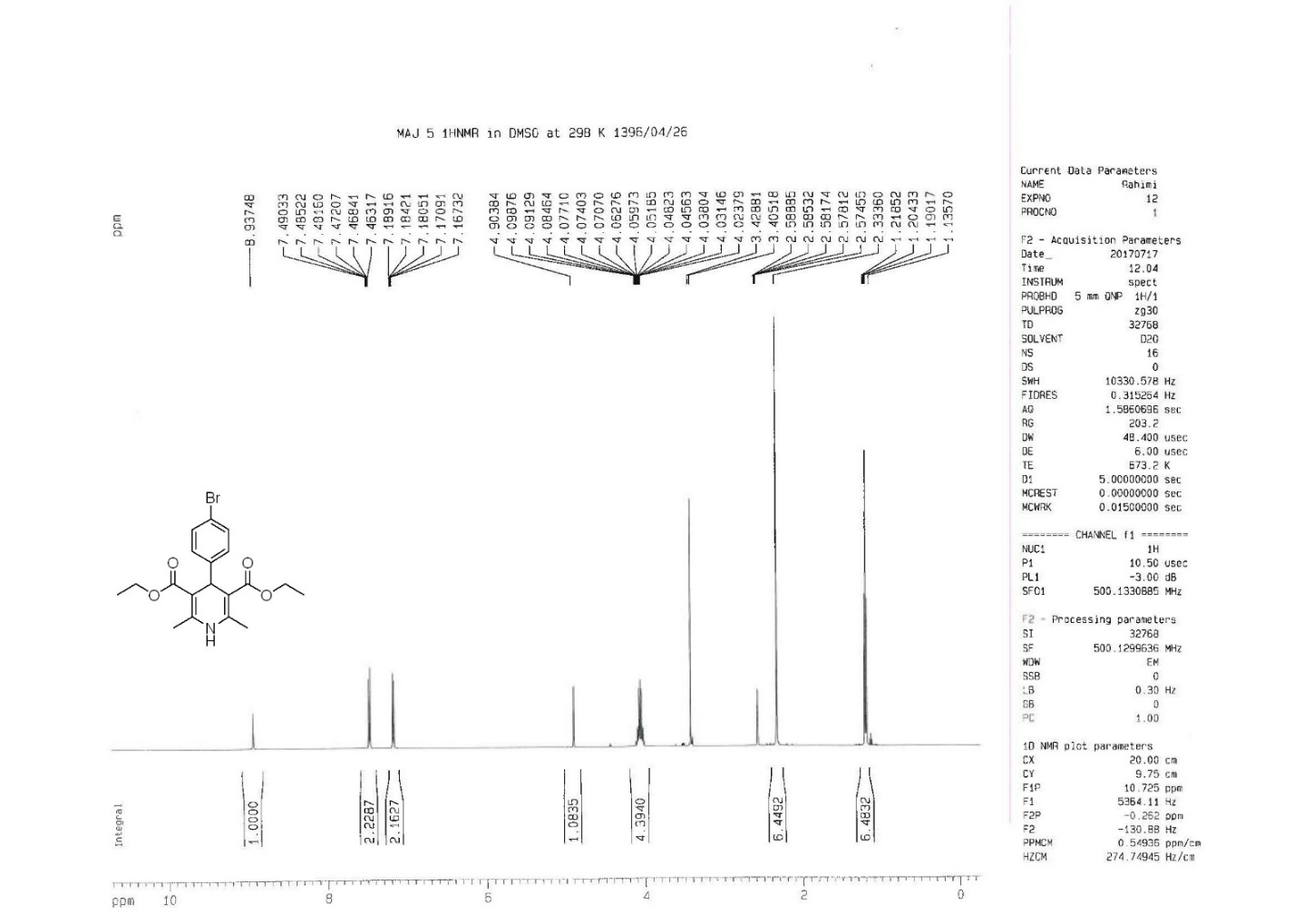


**Figure S21.** ^1^H NMR spectrum of the product **4e.**


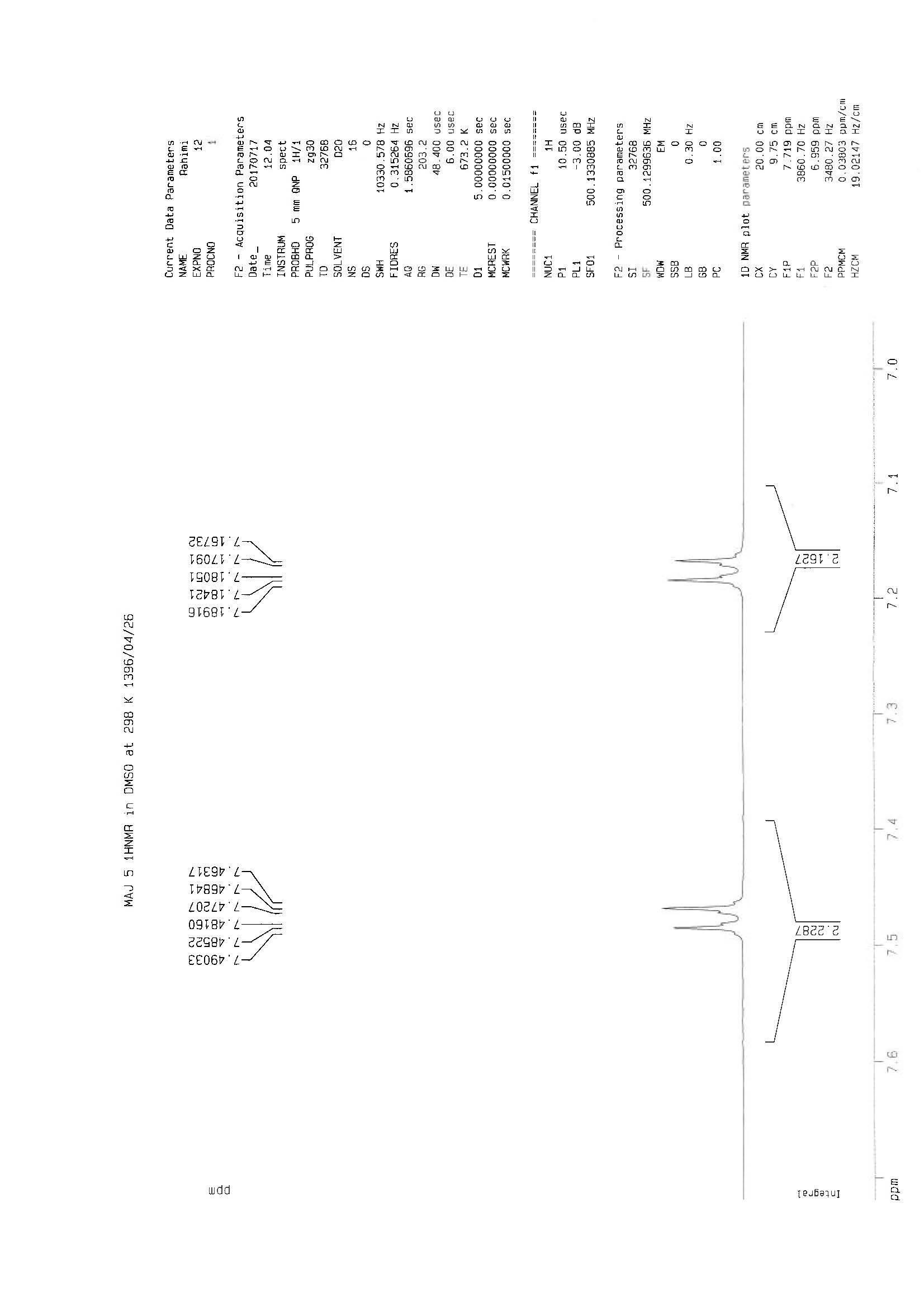


**Figure S22.** ^1^H NMR spectrum of the product **4e.**


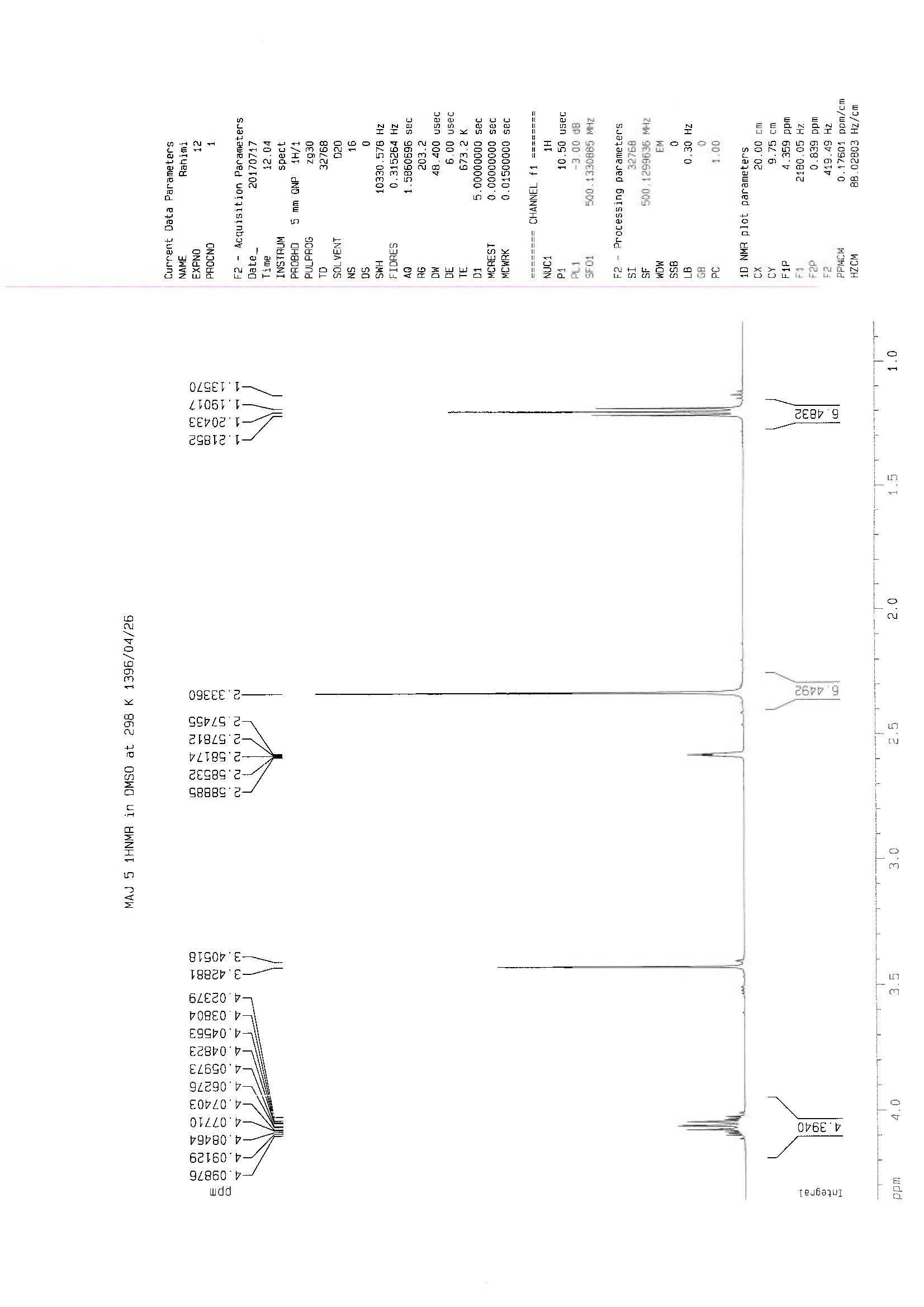


**Figure S23.** ^1^H NMR spectrum of the product **4e.**

**
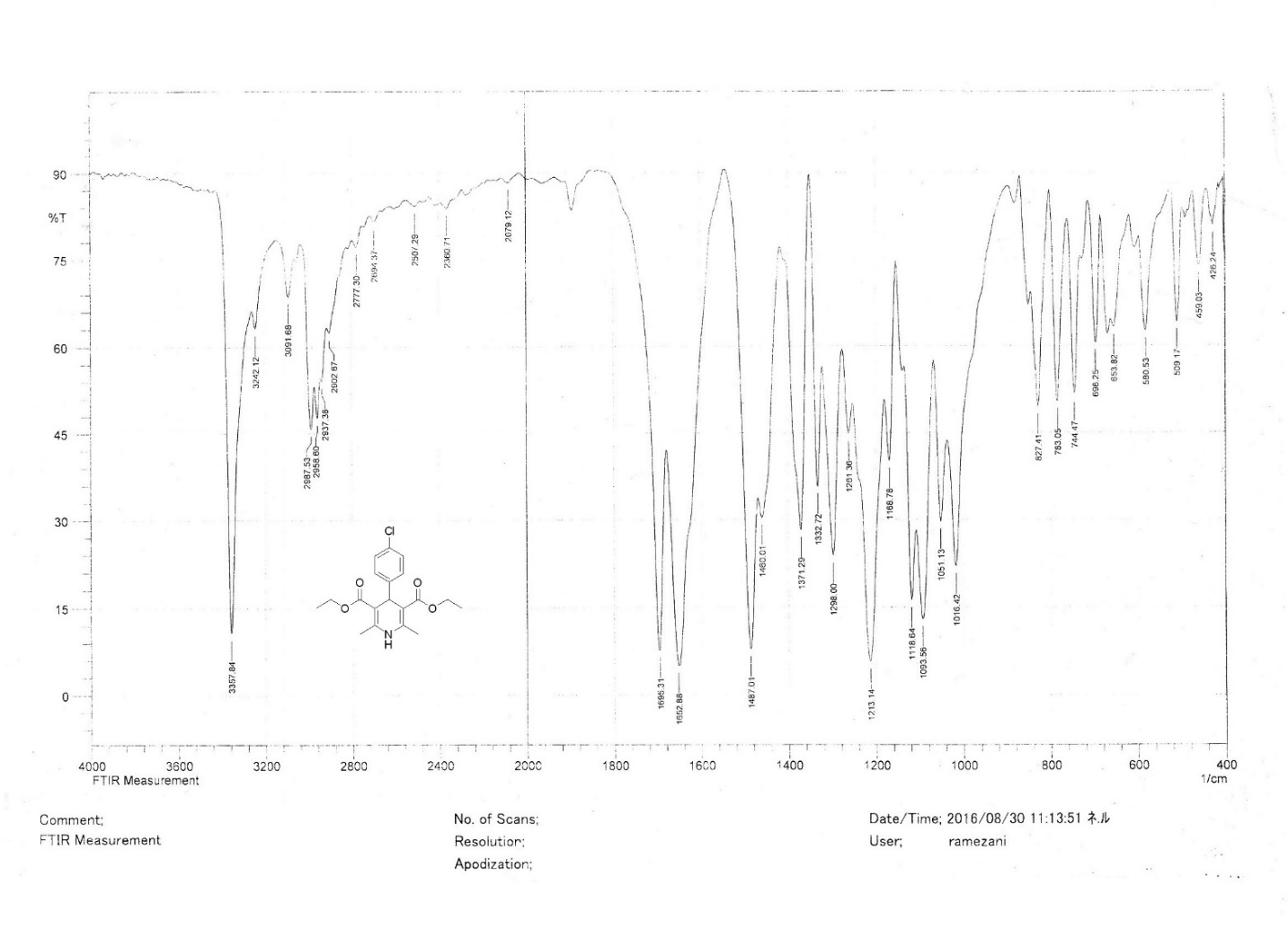
**

**Figure S24.** FT-IR spectrum of the product **4f.**

**
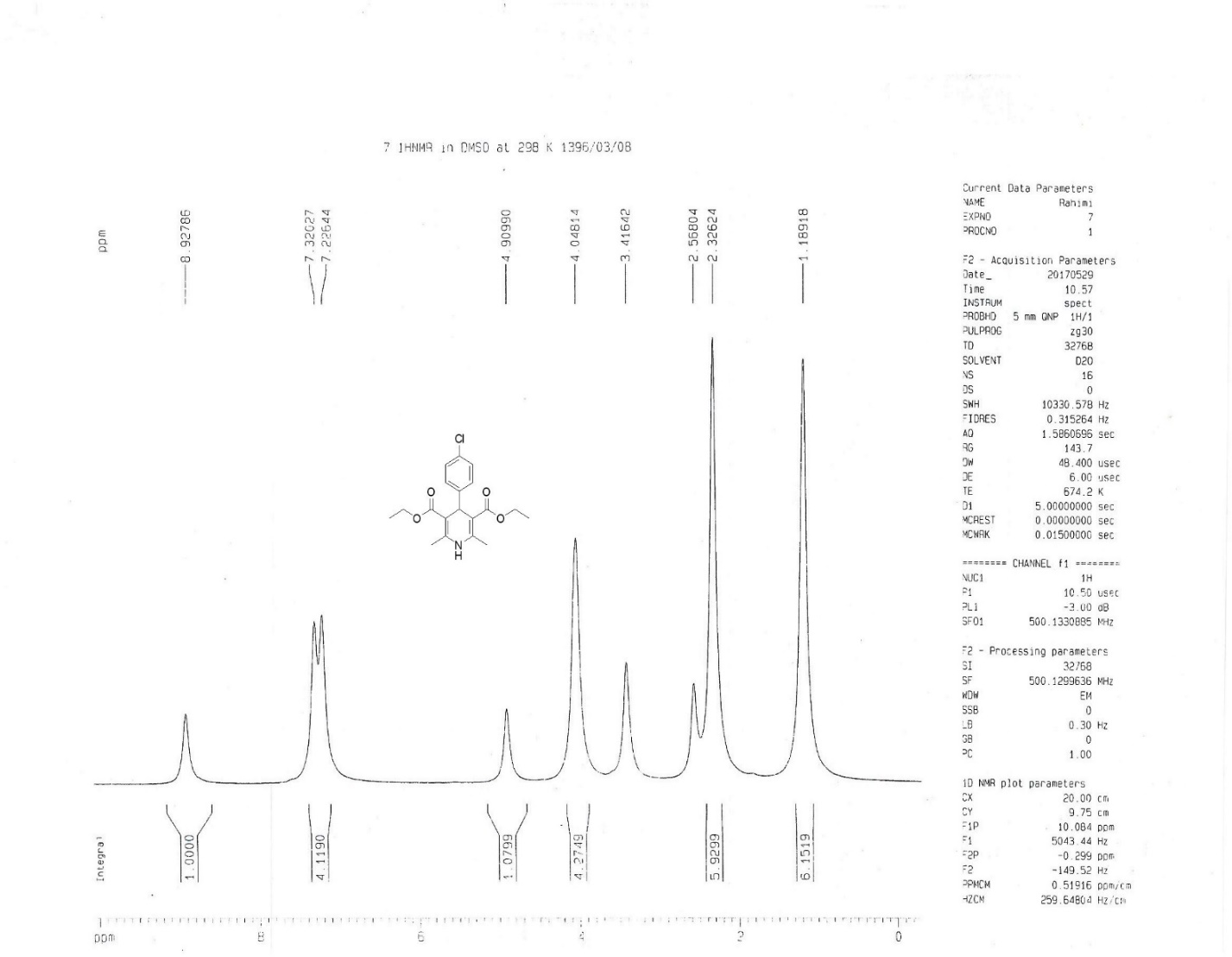
**

**Figure S25.** ^1^H NMR spectrum of the product **4f.**

**
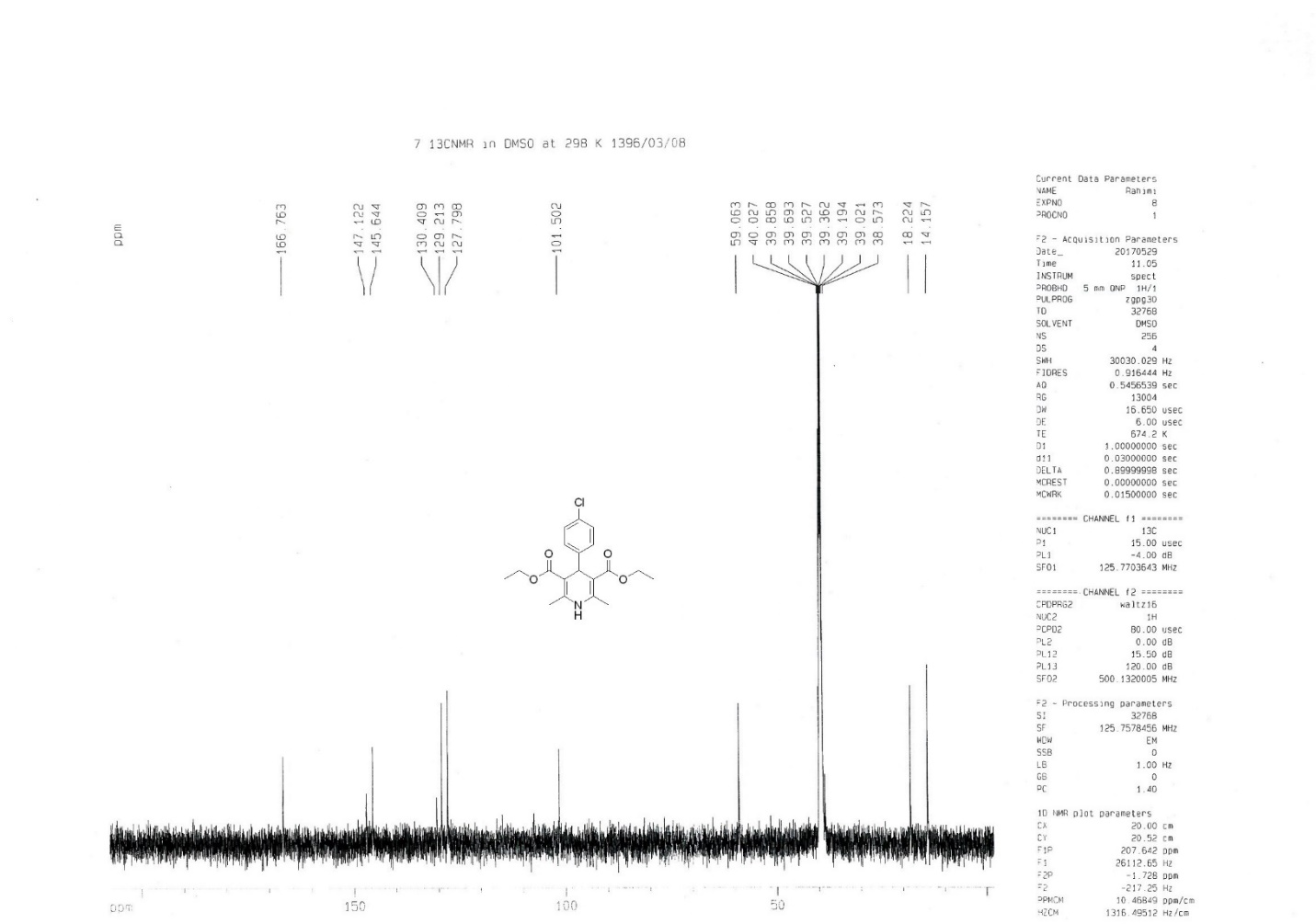
**

**Figure S26.** ^13^C NMR spectrum of the product **4f.**

**
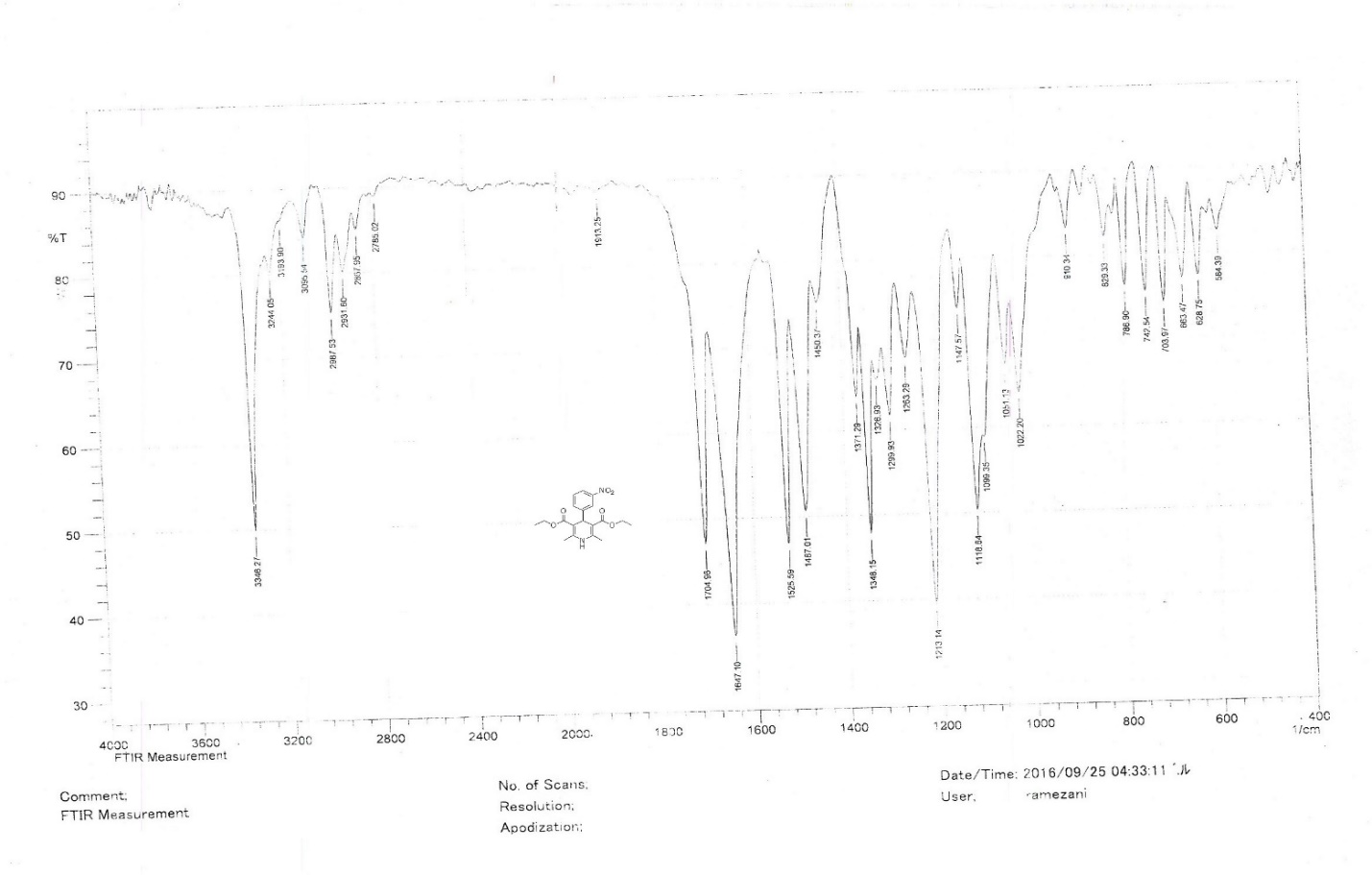
**

**Figure S27.** FT-IR spectrum of the product **4j.**

**
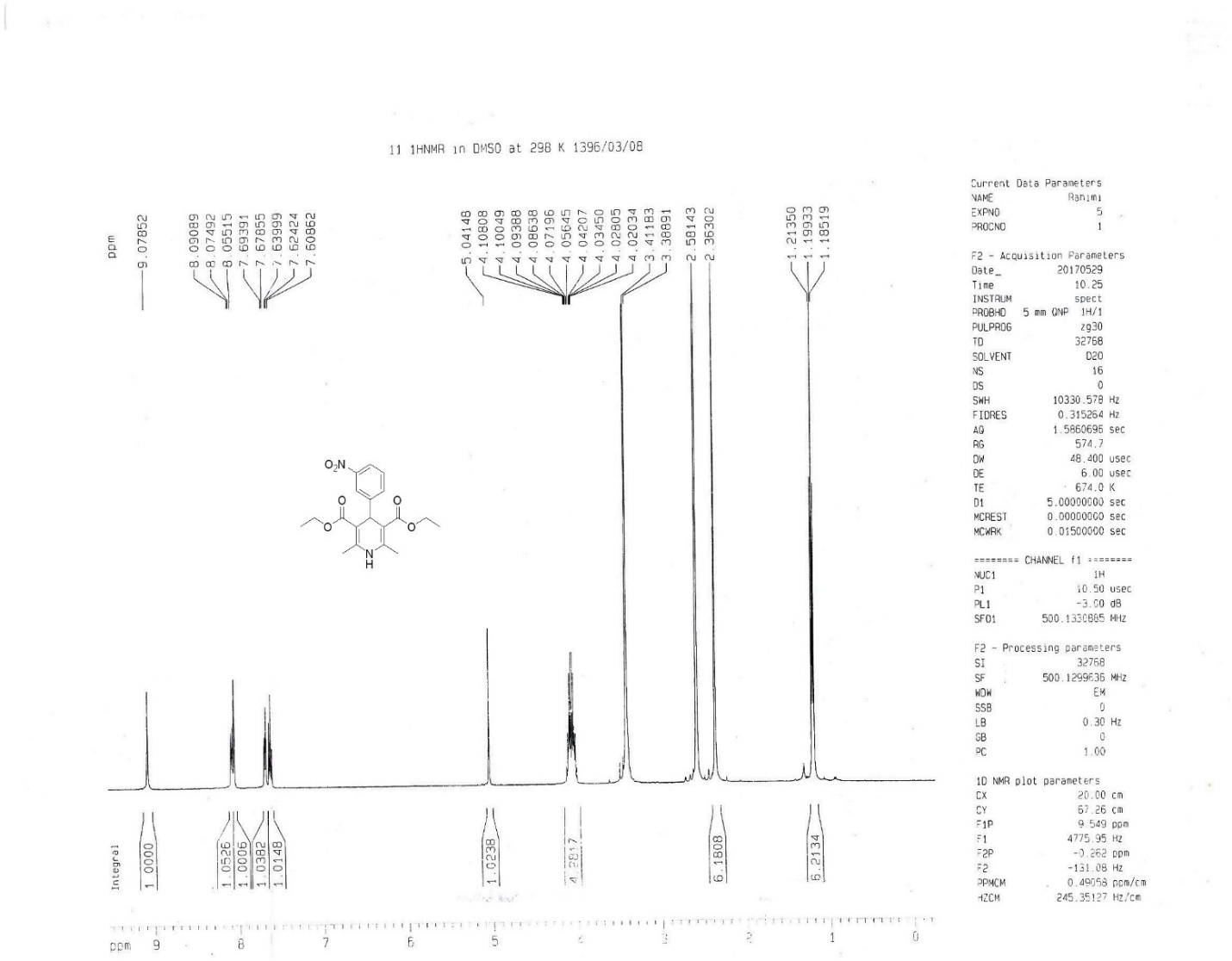
**

**Figure S28.** ^1^H NMR spectrum of the product **4j.**

**
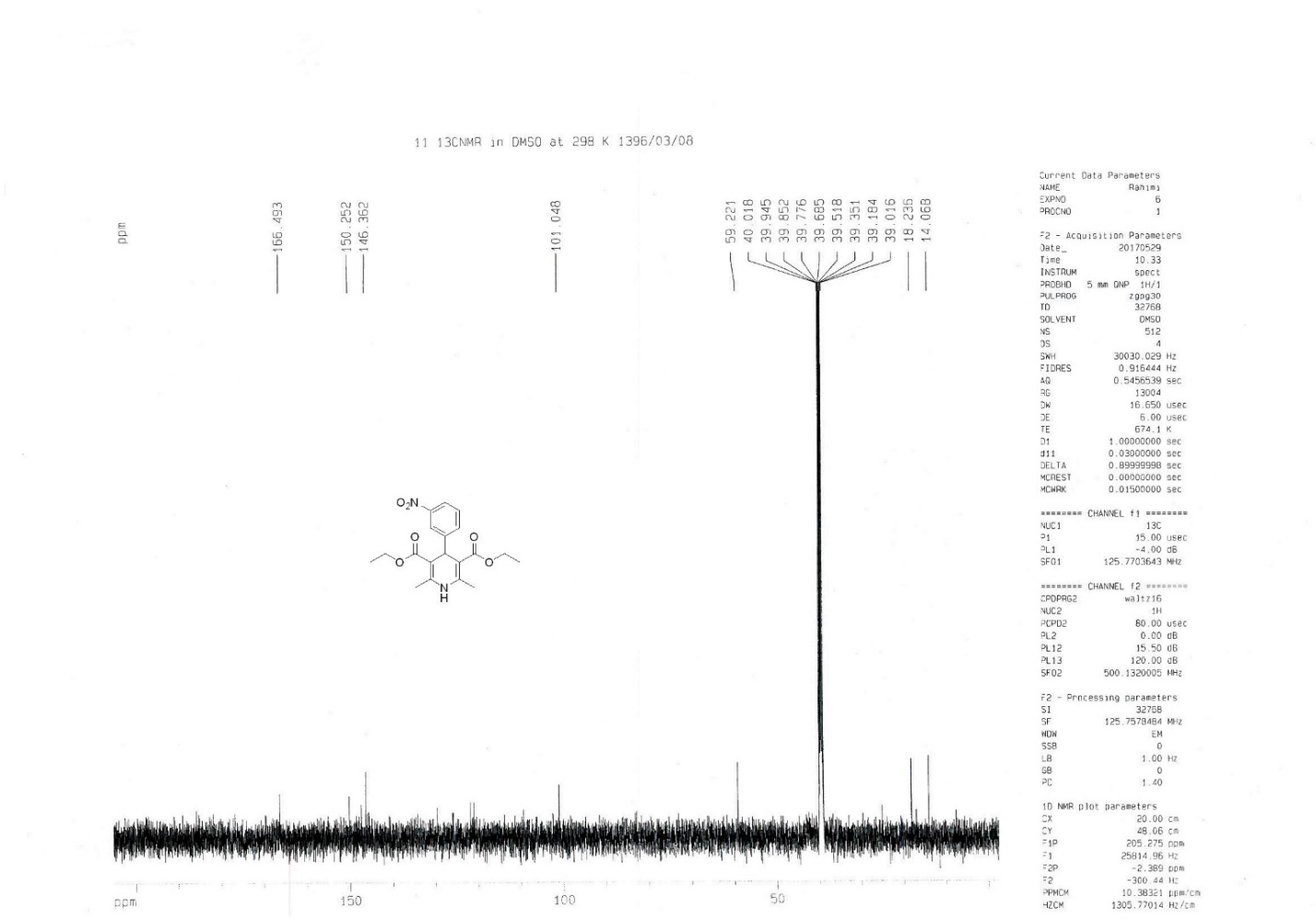
**

**Figure S29.** ^13^C NMR spectrum of the product **4j.**


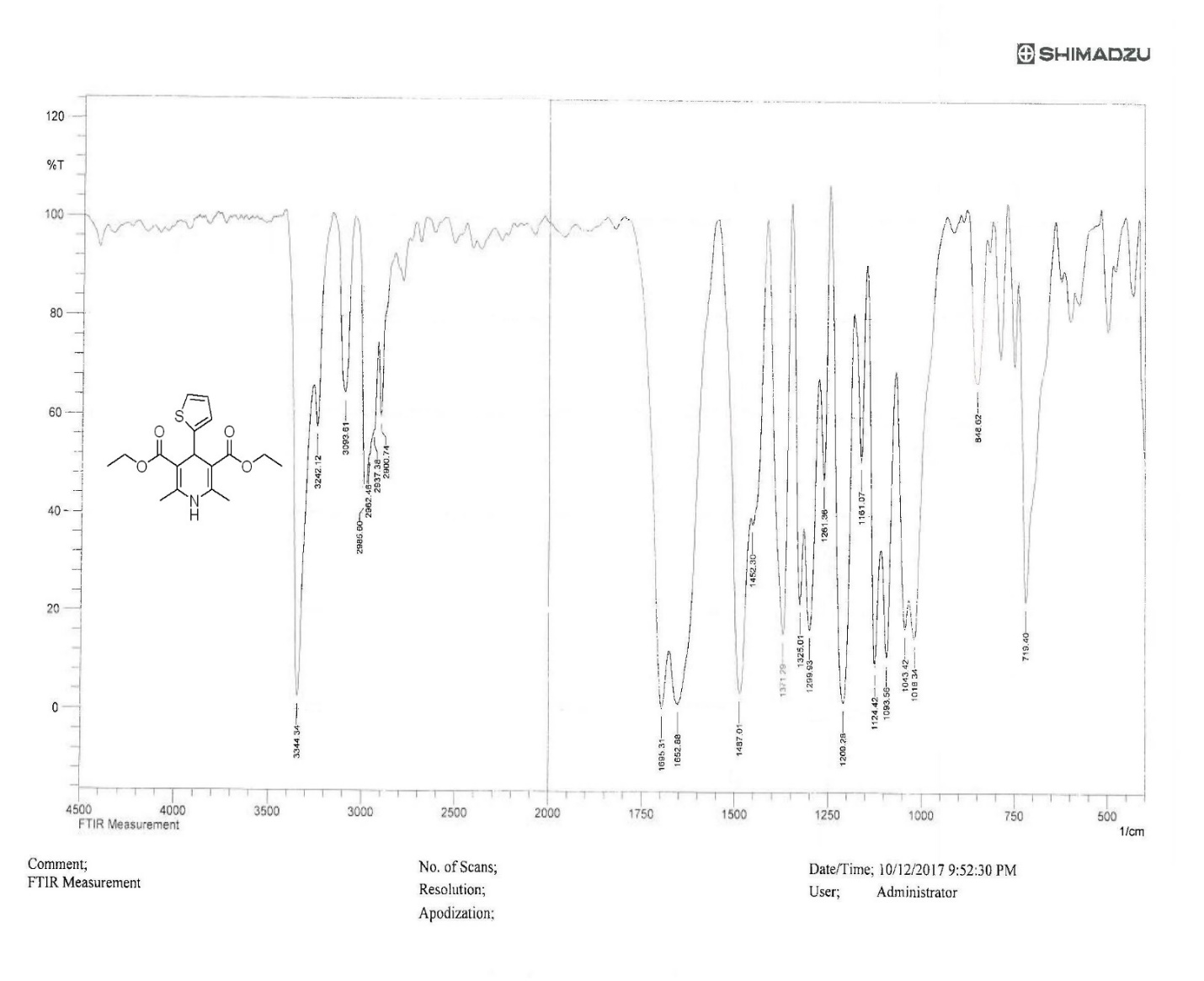


**Figure S30.** FT-IR spectrum of the product **4f.**


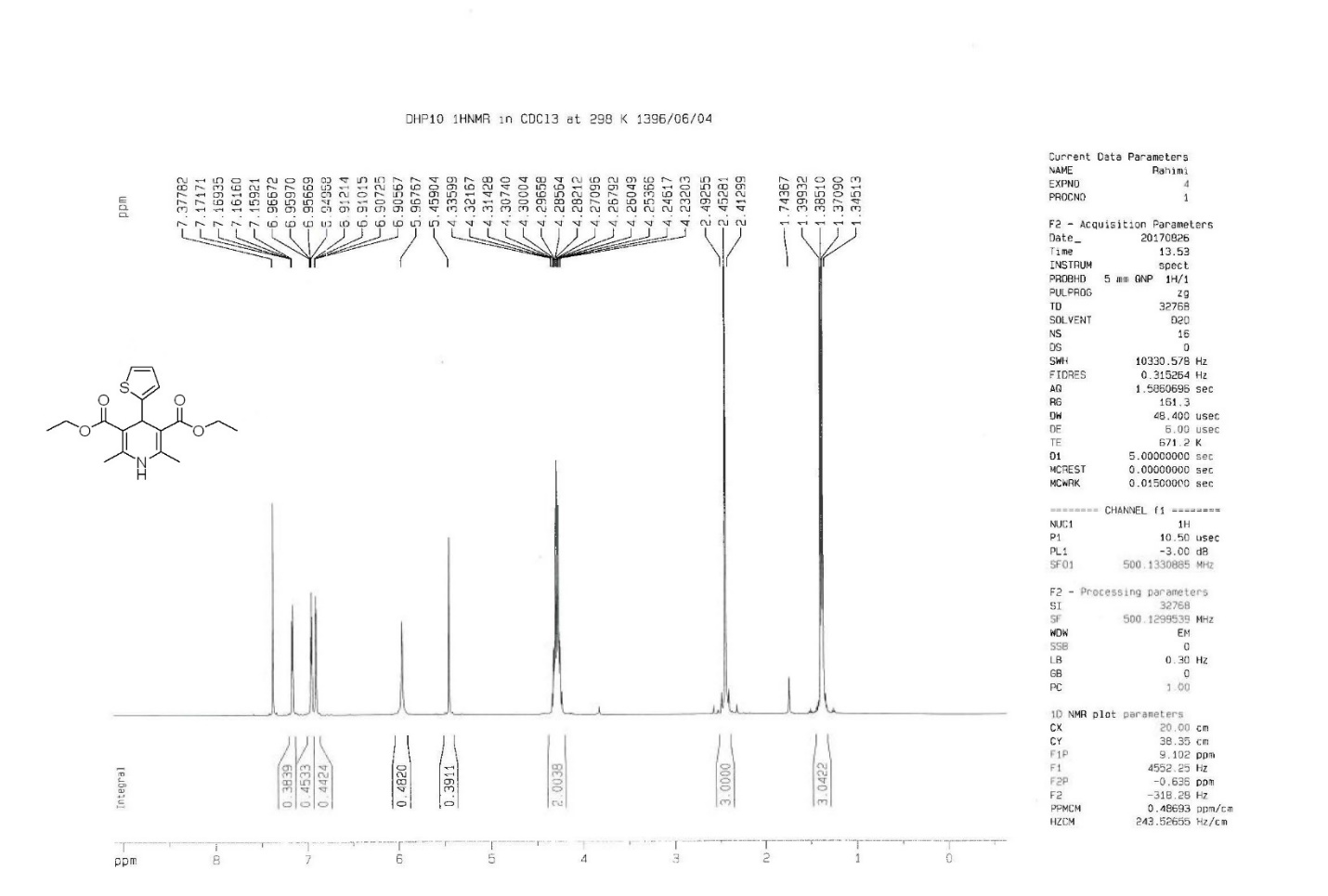


**Figure S31.** ^1^H NMR spectrum of the product **4f.**


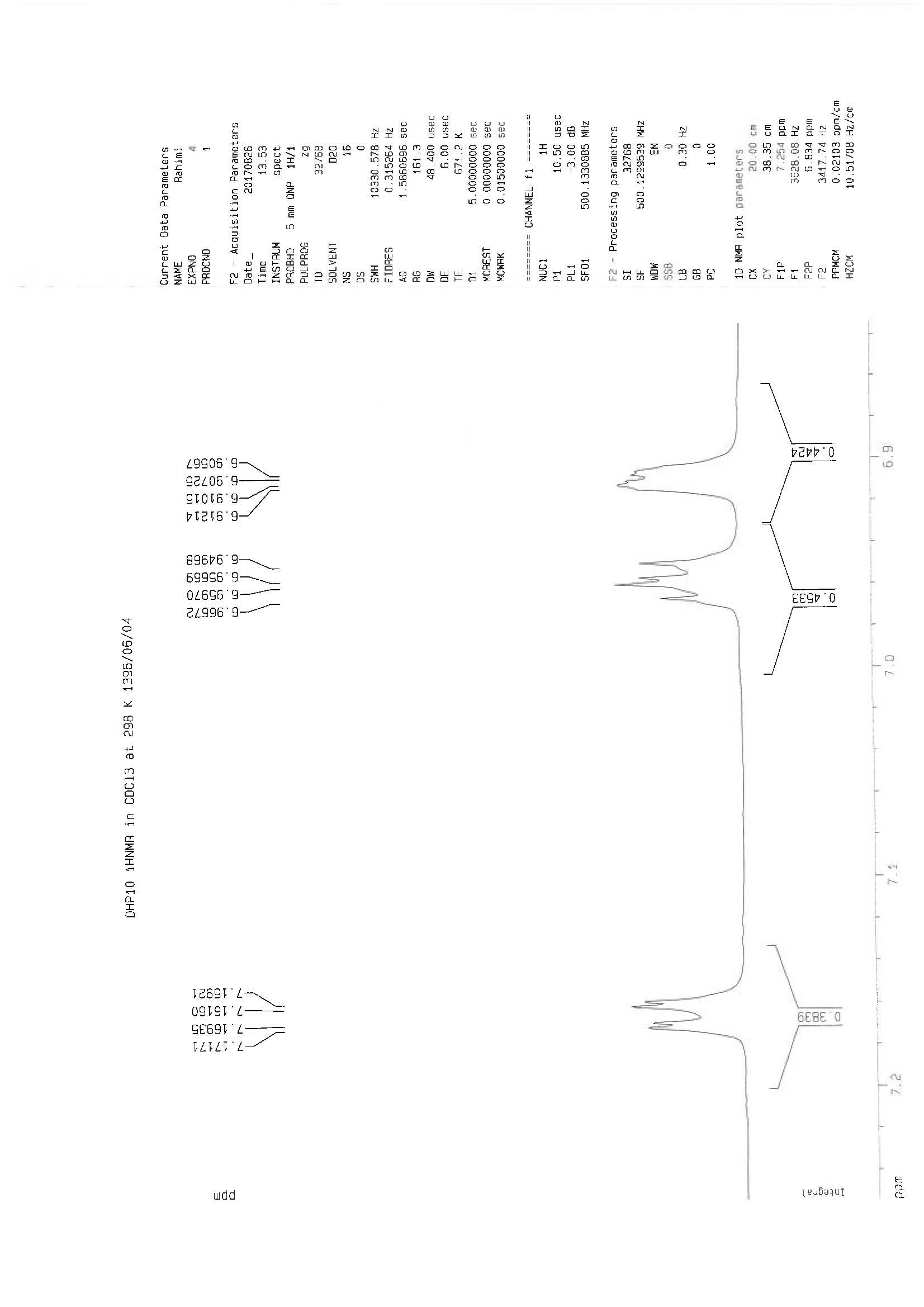


**Figure S32.** ^1^H NMR spectrum of the product **4f.**


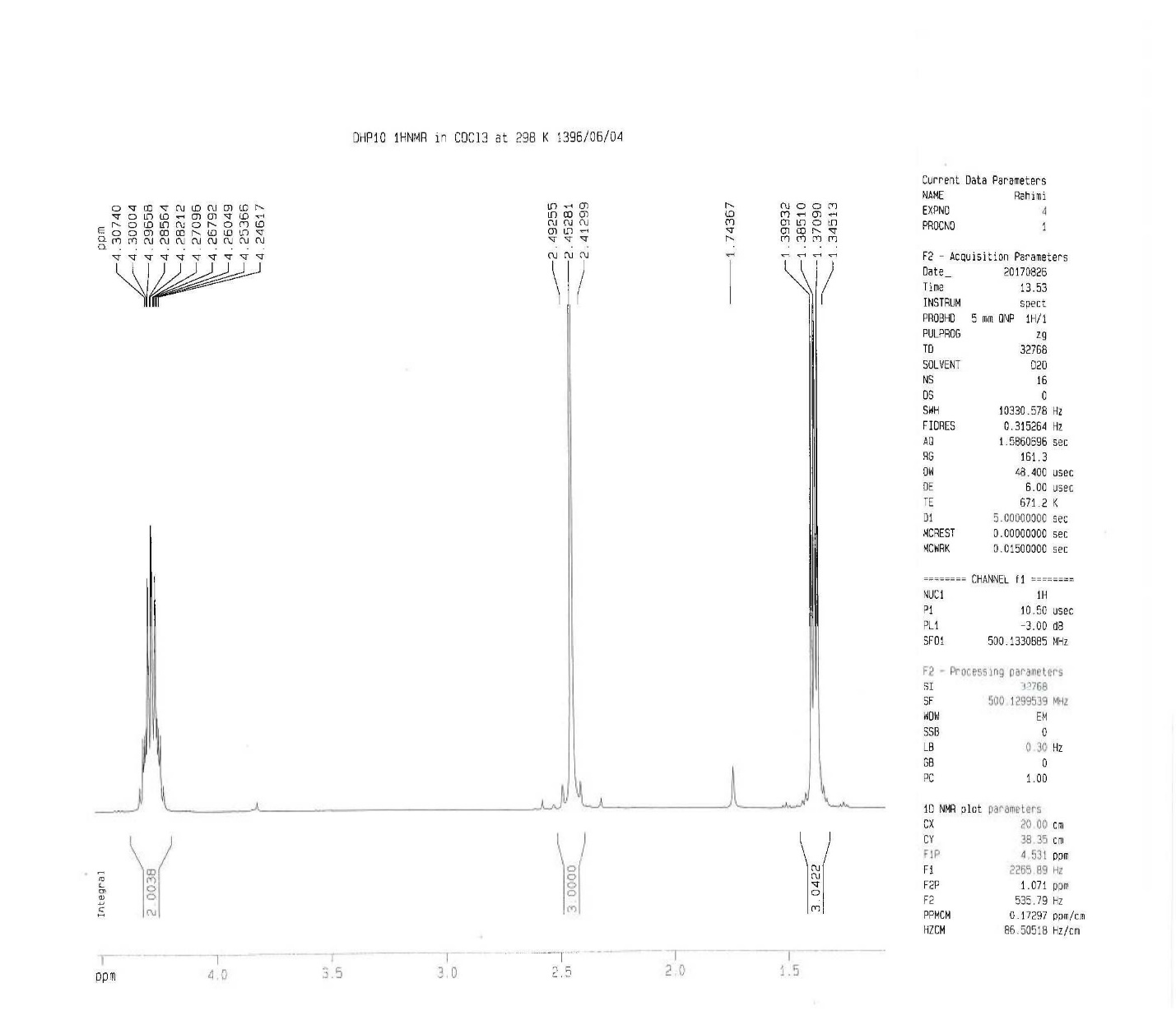


**Figure S33.** ^1^H NMR spectrum of the product **4f.**
